# Parental sleep when their child is sick: A phased principle‐based concept analysis

**DOI:** 10.1111/jsr.13575

**Published:** 2022-04-25

**Authors:** Stephanie Smith, Mary Tallon, James Smith, Charlotte Angelhoff, Evalotte Mörelius

**Affiliations:** ^1^ 60081 School of Nursing and Midwifery Edith Cowan University Perth WA Australia; ^2^ 60081 Perth Children's Hospital Nedlands, Perth WA Australia; ^3^ 1649 School of Nursing Curtin University Perth WA Australia; ^4^ Centre for Precision Health Collaborative Genomics and Translation Group School of Medical and Health Sciences Edith Cowan University Perth WA Australia; ^5^ Centre for Healthcare Resilience and Implementation Science Australian Institute for Health Innovation Macquarie University Sydney NSW Australia; ^6^ 4566 Crown Princess Victoria's Child and Youth Hospital and Department of Biomedical and Clinical Sciences Linköping University Linköping Sweden

**Keywords:** concept analysis, parental sleep, phased principle‐based concept analysis, principle‐based concept analysis, qualitative, sick child

## Abstract

Sleep is a common challenge for parents with sick children and can impact parents' health, wellbeing, and caregiving responsibilities. Despite the vast research around parental sleep when their child is sick, the concept is not clearly defined. A phased principle‐based concept analysis that includes triangulation of methods and quality criteria assessment was used to explore how the concept is described, used, and measured in the current literature. The aim was to analyse and clarify the conceptual, operational, and theoretical basis of parental sleep when their child is sick to produce an evidence‐based definition and to identify knowledge gaps. A systematic literature search including databases CINAHL, Embase, MEDLINE, PsychARTICLES, PsychINFO, Pubmed, Scopus and Web of Science, identified 546 articles. The final dataset comprised 74 articles published between 2005 and 2021 and was assessed using a criteria tool for principle‐based concept analysis. Data were managed using NVivo, and thematic analysis was undertaken. A precise definition is not present in the literature. Various tools have been used to measure parents' sleep, as well as exploration via interviews, open‐ended questions, and sleep diaries. The terminology used varied. Parental sleep when their child is sick is interrelated with other concepts (e.g., stress). A recommended definition is offered. A conceptual understanding of parental sleep when their child is sick will help to guide translational research and to conduct studies critical to clinical practice and research. Future research includes developing a measurement tool for parental sleep when their child is sick to be used in study design and future interventions.

## 1 INTRODUCTION

Sleep is a physiological process and one of the most fundamental human needs (Zhu et al., 2018). It is essential for health, wellbeing, and performance (Lee‐Chiong, 2008). According to Merriam Webster, sleep is “the natural, easily reversible periodic state of many living things that is marked by the absence of wakefulness and by the loss of consciousness of one's surroundings, is accompanied by a typical body posture (such as lying down with the eyes closed), the occurrence of dreaming, and changes in brain activity and physiological functioning, is made up of cycles of non‐REM sleep and REM sleep and is usually considered essential to the restoration and recovery of vital bodily and mental functions” (Merriam‐Webster, 2021).

Parents' sleep can be affected for multiple reasons (Palmstierna et al., 2008). A child who is sick can demand more supervision and care, making sleep disturbance a common challenge among parents of sick children (Stickland et al., 2016). Concerns about the child's diagnosis, treatment, and care can be a significant source of worry and anxiety (Cousino & Hazen, 2013; Stremler et al., 2011), adding to daily hassles and therefore affecting sleep quality. Sleep not only affects parents' wellbeing but also their child's care as symptoms of sleep loss may limit their parents' ability to meet their child's needs (Angelhoff et al., 2015). Negative parenting behaviours, such as high stress, low self‐efficacy, and greater irritability in parent–child interactions have been associated with sleep loss. It remains important for parents to be able to rest and sleep so they can restore energy to be able to meet their child's health needs. Sleep is therefore important for the parents' own physical and emotional health, their ability to cope with the illness event, support their child and other family members, participate in decision‐making and to maintain relationships (Stremler et al., 2011).

Despite the impact that parental sleep can have on the parents themselves, the sick child, and the whole family, parental sleep when their child is sick is a fairly recent research area that requires more attention (Angelhoff et al., 2017; Mörelius & Hemmingsson, 2013). An integrative review on parents' experiences of sleep when they stay overnight with their hospitalised child supports the need to clarify the meaning of sleep among parents (Løyland et al., 2019). Considering the growing literature in this field, it is important to define a common language to use when performing research in this area. This will increase the possibility to compare studies, perform meta‐analyses, draw conclusions, and develop and provide evidenced‐based care in the future.

Principle‐based concept analysis (Penrod & Hupcey, 2005) reviews the strengths and limitations of the present state of a concept in the scientific literature and can assist the clarification and development of the concept parental sleep when their child is sick. It is the analysis of a concept according to four broad philosophical principles: epistemological, pragmatic, linguistic, and logical (Smith & Mörelius, 2021). Epistemology is concerned with how the concept is defined and differentiated within the literature (Waldon, 2018). The pragmatic principle considers the concept's usefulness and whether it has been operationalised (Smith & Mörelius, 2021). The linguistic principle evaluates the consistency of use and meaning of a concept within the literature, and the context is also considered (Waldon, 2018). The logical principle considers the theoretical integration of the concept with other related concepts (Ruel & Motyka, 2009). Clearly defined conceptual boundaries are essential to prevent loss of meaning caused by conflicting attributes when positioned with other concepts in a theoretical framework (Ruel & Motyka, 2009). A principle‐based concept analysis reduces the data (literature) through initially reviewing and summarising the four principles (Smith & Mörelius, 2021). The conceptual components (the construction of the concept) are then explored via the preconditions (phenomena or events that precede an instance and that influences the concept), characteristics/attributes (frequent words or expressions used to describe the experience of the concept), and outcomes (the consequences that follow the occurrence of the concept) (Waldon, 2018). The final product is a theoretical definition based on integrating the summaries of the four principles and the conceptual components or highlighting an existing definition that covers these aspects.

The purpose of this study was to analyse and clarify the concept of parental sleep when their child is sick by exploring how it is described and used within the literature and to introduce a theoretical definition.

## 2 METHODS

### 2.1 Study design

A phased principle‐based concept analysis on parental sleep when their child is sick, according to Smith and Mörelius (2021), was followed. This comprehensive approach to conducting a principle‐based concept analysis is systematic and enhances transparency, rigour, and replicability. Table 1 outlines the three phases followed.

**TABLE 1:** A phased approach to conducting a principle‐based concept analysis (reprinted with permission)

Phase	Stage 1	Stage 2	Stage 3	Stage 4
1: Preparation	Determine the concept of interest	Develop a protocol	Systematic literature search	Screen articles
2: Analysis	Initial note‐taking	Adapt and pilot test the quality criteria tool	Quality criteria assessment	Integrate data
3: Results	Quality criteria findings of the included articles	Summative conclusions of the four principles	Conceptual components	Theoretical definition

#### 2.1.1 Phase 1: preparation phase

Preparing a phased principle‐based concept analysis included determining why parental sleep when their child is sick needed to be explored, developing a protocol outlining the process, conducting a systematic search, and screening the articles.

The team's expertise was also considered. A varied research team aids to advance the transference of knowledge across disciplines (Smith et al., 2018), which applies to the principle‐based concept analysis and promotes exploring multidisciplinary perspectives in understanding a concept (Penrod & Hupcey, 2005). The research team included a wide range of disciplines from health psychology, implementation science, neonatology, and paediatrics.

##### Phase 1; stage 1: determine the concept of interest

The concept of interest was parental sleep when their child is sick. The terms “parental sleep when their child is sick” and “concept” are used interchangeably in this review. This concept has been identified in the literature without a precise definition (Løyland et al., 2019) and is an area that requires further consideration (Angelhoff et al., 2017; Mörelius & Hemmingsson, 2013).

##### Phase 1; stage 2: develop a protocol

An a priori protocol was developed to outline the databases to search, the inclusion/exclusion criteria, data extraction, quality criteria, and synthesis. All of the authors were provided with the protocol.

##### Phase 1; stage 3: systematic literature search

To determine the scope and range of the literature, a systematic review search was conducted based on the Centre for Reviews and Dissemination guidelines from the University of York, UK (CRD, 2009; Dehghani et al., 2018).

An experienced research librarian was consulted on the databases and keywords to use as recommended (Aromataris & Munn, 2017). This concept constitutes several important keywords. Keywords were used in all databases, and when possible, controlled terms were used. Box 1 details the variation of the keywords searched.

BOX 1 **Search strategy**

**Table:** 

**Core databases (CINAHL, PsychInfo, PsychArticles, Embase, Medline)** Parent* mother* father* “sleep quality” “quality of sleep” “sleep disrupt*” “sleep disturb*” “sick child*” “ill child*” “sick adoles*” “ill adoles*” 1 OR 2 OR 3 4 OR 5 OR 6 OR 7 8 OR 9 OR 10 OR 11 12 AND 13 AND 14 **Supplementary databases (Pubmed, Scopus, Web of Science):** Parent* OR mother* OR father* AND “sleep quality” OR “quality of sleep” OR “sleep disrupt*” OR “sleep disturb*” AND “sick child*” OR “ill child*” OR “sick adoles*” OR “ill adoles*”

One author conducted the searches, and the results were exported to Endnote (The EndNote Team, 2013) to manage the process. The searches were limited to English and academic journals/peer‐reviewed journals if available. No time restriction was used in order to capture relevant studies and an overview of the concept over time (Zhu et al., 2018). In paediatric nursing, it is important to have a generic perspective and to find commonalities and support possibilities that suit parents irrespective of the child's disease or diagnosis (Leeman et al., 2016; Meltzer & Moore, 2008). Therefore, we did not restrict the search to particular conditions. The initial search was carried out in June 2019 and updated in September 2021. Table 2 outlines the criteria and the databases searched.

**TABLE 2:** Inclusion/exclusion criteria and database results

Inclusion	Exclusion	Database results	Total
Peer‐reviewed English language Articles on parental sleep including alongside other populations (e.g., children's sleep, healthcare professionals' perceptions) Any methodology All years All health conditions	Theses Reviews Conceptually unrelated articles Abstracts only	CINAHL Embase Medline PsychARTICLES PsychInfo PubMed Scopus Web of science Other sources Total	7 42 35 0 22 42 303 50 45 546

##### Phase 1; stage 4: screen articles

**FIGURE 1:**
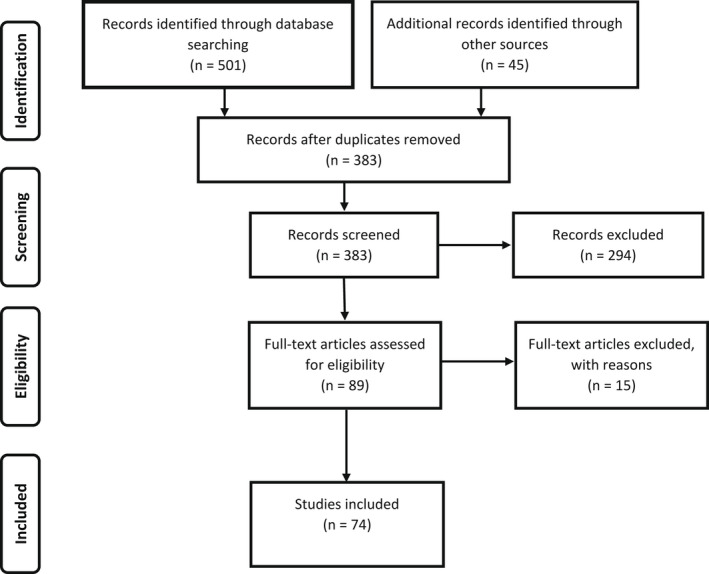
PRISMA flow diagram of the screened studies

Figure 1 provides the screening process followed using the Preferred Reporting Items for Systematic Reviews and Meta‐Analyses (PRISMA). To assure quality, two authors simultaneously and independently carried out the screening process by initially reading the title and abstracts and sequentially excluding records according to the inclusion and exclusion criteria. When inclusion or exclusion could not be determined based on title and abstract, the article was moved forward for full‐text screening. Hand searches included reviewing two journals chosen based on the number of database search results, Google Scholar, and reference lists of the final articles. Full‐texts on the resultant articles were read to determine if they met the inclusion criteria. Eleven articles resulted in different opinions/unsure of including and the final decision for inclusion was made by a third author.

### 2.2 Analysis

#### 2.2.1 Phase 2: analysis phase

Data analysis of the included articles followed the six stages of Braun and Clarke's (2006) thematic analysis (familiarisation with the data, coding, generating initial themes, reviewing themes, defining and naming themes, writing up) and are incorporated throughout the phased principle‐based concept analysis.

##### Phase 2; stage 1: initial note‐taking

Each article was reviewed in‐depth. Familiarising included reading the article, and notes or text were highlighted on the article itself on anything of interest. Parental sleep terms or associated terms/characteristics were highlighted on the article. Notes were made separately for each of the four principles and consisted of (1) Epistemology, if any definitions were made or part definitions about the concept, characteristics used, terms and associated terms used, (2) Pragmatic, tools and methods used to measure/explore the concept, (3) Linguistics, notes on consistency and use of terms, (4) Logical, theories mentioned around the concept. Notes were also made for the conceptual components identified in the articles that are covered in more detail in Phase 3; Stage 3.

##### Phase 2; stage 2: adapt and pilot test the quality criteria tool

To enhance reliability and validity, three authors (with one author also acting as facilitator) independently reviewed six articles against the quality criteria for a phased principle‐based concept analysis for comparison of answers and adapting the quality criteria tool (Appendix S1, Quality criteria) to the concept. Articles included in the pilot test covered various methods (qualitative, quantitative, mixed‐methods), research conducted in different countries and disciplines to ensure a variety of research was reviewed. The articles and a spreadsheet with the quality criteria were emailed to the researchers, and the articles were split and reviewed over three online meetings. The guidelines for the pilot test involved reading each article twice, completing the quality criteria with “Yes”, “Partly” or “No” (Quality Criteria scores: Yes ‐ 2 points, Partly ‐ 1 point, No ‐ 0 points) for the questions and providing evidence for the decision on the spreadsheet for comparison, and providing a score for each principle and an overall rating for the article. Before each meeting, the facilitator collated the results of the reviews, and the quality criteria spreadsheet was updated based on previous discussions and emailed to the researchers. Group discussions also enabled familiarisation of the data and reviewing the findings against the tool. Reviewing a small number of articles at each meeting enabled any queries to be covered to aid the following meetings, comparison of results, and to adapt the tool as required to the concept.

##### Phase 2; stage 3: quality criteria assessment

Further familiarisation occurred with rereading the article and completing the quality criteria for a phased principle‐based concept analysis tool on a spreadsheet as outlined in phase 2; stage 2. This was an iterative process reviewing the article and previous notes made. Appendix S1 shows the Smith and Mörelius (2021) quality criteria tool for a phased principle‐based concept analysis adapted to the concept, parental sleep when their child is sick.

##### Phase 2; stage 4: integrate data

Braun and Clarke's (2006) stages of coding, generating initial themes, and reviewing themes are included here. Key points of the article were coded deductively in NVivo (QSR International Pty Ltd, 2018) to the principle it most closely associated with. For example, Epistemological – text was coded to definitions/part definitions, terms/characteristics used, associated terms (e.g., stress, depression). Pragmatic – if the research was useful, applicable, and appropriate to the concept, tools used to measure/explore parents' sleep (e.g., questionnaires, diaries), and recommendations. Linguistic – consistency/inconsistency of terms. Logical – any noted or lack of boundaries with other concepts and models/theories mentioned. Coding also included references to the conceptual components, preconditions, and outcomes. The characteristics were captured and coded to the epistemological principle. During coding, the initial notes were referred to, and key and associated terms were checked off to ensure all terms were captured and acted as a validity check. The quality criteria notes for each principle were also referred to as this provided an overview of each article. Initial themes were generated inductively within each principle. Once all the articles had been coded, the initial themes were reviewed against the dataset and combined and defined. This involved critically examining the concept of parental sleep when their child is sick according to the clarity of definition (epistemology), applicability of the concept (pragmatic), consistency in use and meaning (linguistic), and differentiation of the concept from related concepts (logical) (Bicking Kinsey & Hupcey, 2013). Regular meetings were held throughout the analysis process with two of the authors to explore the data and to discuss themes. Final themes were reviewed with all the authors.

## 3 RESULTS

### 3.1 Phase 3: results phase

The quality criteria findings, summative conclusions of the four principles, the conceptual components and the theoretical definition of parental sleep when their child is sick are presented. Braun and Clarke's (2006) fifth stage of defining and naming themes and sixth stage of writing and presenting the findings are presented in this section under each principle. When more than 10 articles are cited, this number is stated (e.g., 34 out of 56 articles) with an example reference for readability purposes. Where multiple points are presented in one sentence, a reference is provided for each point. The complete list of these references is provided in Appendix S3, Table of references, with a point number that section refers to (e.g., see Appendix S3, Table of references, point 1). The example references have been selected based on recently published articles or to ensure various articles are cited throughout the text.

#### Phase 3; stage 1: quality criteria findings of the included articles

The final dataset consisted of 74 articles. Appendix S2 highlights the literature overview. Table 3 shows a visual summary of the findings (noted as a useful representation of qualitative research (Smith et al., 2021; Smith et al., 2018)) to highlight the articles to the four principles and the quality scores of the included articles. No studies were excluded because of the scores since the purpose of this study was to define a concept.

**TABLE 3:**
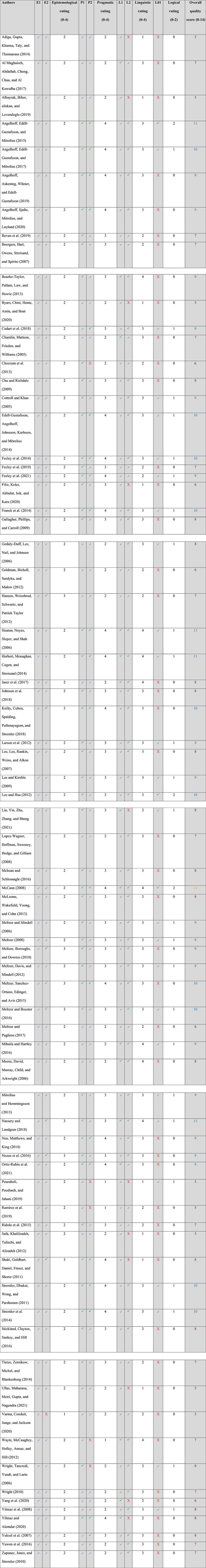
Quality scores of the included articles

Note

**Key:** 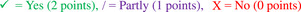

**E1:** Is ‘parental sleep when their child is sick’ defined?

**E2:** Is ‘parental sleep when their child is sick’ differentiated/distinguished from other concepts (e.g., child’s sleep, controls, sleep diseases, exhaustion, depression, mood, and other biopsychosocial health)?

**P1:** Is *‘parental sleep when their child is sick’* useful and applicable (e.g., researching *‘parental sleep when their child is sick’* and/or applies to *‘parental sleep when their child is sick’* through the study purpose/aims of the research/identification of knowledge gaps, findings, and recommendations) within health disciplines (e.g., beneficial to healthcare, clinical practice, or research)?

**P2:** Has the concept *‘parental sleep when their child is sick’* been appropriately measured/explored and evaluated (e.g., ethical considerations, sample, measures used, policies/interventions developed)?

**L1:** Is *‘parental sleep when their child is sick’* or the language, or the key attributes and characteristics around *‘parental sleep when their child is sick’* identified and used **consistently** within the whole article?

**L2:** Is *‘Concept’* or the language or the key attributes and characteristics around *‘parental sleep when their child is sick’* used **appropriately** within the context of the article?

**LO1:** Does *‘parental sleep when their child is sick’* hold its boundaries through theoretical integration with other related concepts (e.g., in theories, models, or frameworks)?

The overall quality scoring scale

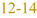
: Provides *significant* information to advance understanding of ‘parental sleep when their child is sick’.

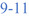
: Provides *good* information to advance understanding of ‘parental sleep when their child is sick’.

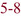
: Provides *some* useful information to advance understanding of ‘parental sleep when their child is sick’.

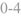
: Provsides *minimal* information to advance understanding of ‘parental sleep when their child is sick’.

#### Phase 3; stage 2: summative conclusions of the four principles

Each principle is reviewed and discussed under the following principles subthemes. Table 4 provides a summary of the principles findings.

**TABLE 4:** Principle summaries

Principle	Subthemes
Epistemological	Poor sleep quality Prolonged sleep latency Frequent sleep disturbance Shorter sleep duration
Pragmatic	Subjective measurements Subjective explorations Objective measures Study participants
Linguistic	Variety of terms Overlapping descriptions Interchangeable terms
Logical	Interrelated concepts Differentiation Theoretical integration

#### Epistemological

The epistemological principle focuses on what is known about a concept of interest (Smith & Mörelius, 2021). The definition of a concept and whether it is clearly defined and differentiated from other concepts in the literature was reviewed.

There were no explicit definitions of parental sleep when their child is sick within the literature. However, many implicit meanings were present, which contribute to the identification of the key characteristics defining this concept. Distinguishing the concept from other related concepts such as sleep, stress, exhaustion, child sleep, and controls, aided to review whether parental sleep when their child is sick is different from other concepts or related concept definitions.

The limited definitions provided included, Chu and Richdale (2009) referring to Stores (2001) description of sleep as a reversible state of reduced awareness and responsiveness to the environment. Angelhoff et al. (2019) noted Lee‐Chiong's (2008) description of sleep as a basic need essential for health, well‐being, and performance. The importance of sleep for recovery was also emphasised by Angelhoff et al. (2015). Commonly, measurable aspects of sleep around time were referenced, especially in terms of sleep patterns, including bedtime, sleep onset latency, night waking, morning wake time, and overall sleep quality (Meltzer et al., 2010). Adequate sleep was defined as falling asleep within 5–10 min after the light is out, night‐time total sleep time of no less than 7 h per day, staying asleep for at least 90% of the time in bed, and feeling refreshed after awakening (Lee & Hsu, 2012; Lee & Kimble, 2009). Parental sleep when their child is sick was presented as a contrast to sleep descriptions as highlighted by the following subthemes, and often the differences are emphasised by comparisons with parents of typically developing children/controls. Parents of typically developed children (e.g., Chu & Richdale, 2009) or controls (e.g., Albayrak et al., 2019) were also described as age‐matched in the general population (Lee & Kimble, 2009), parents of healthy children (e.g., Wright, 2010), the children having no known developmental or chronic health condition (Keilty et al., 2018) or physical disability interpreted as a neurodevelopmental disorder and a physical functional problem (Wright et al., 2006) (see Appendix S3, Table of references, point 1). The overlap of the terms will be discussed in the linguistic principle.

##### Poor sleep quality

Sleep quality was frequently explored in the literature and portrayed an overview of the sleep experience and was part of the research aims for 27 out of 74 articles (e.g. Yilmaz & Alemdar, 2020) (see Appendix S3, Table of references, point 2). Despite studies often omitting a definition of sleep quality (Albayrak et al., 2019; Chu & Richdale, 2009; Cottrell & Khan, 2005; Liu et al., 2021; Pouraboli et al., 2019; Yilmaz & Alemdar, 2020), it has been described as how well a person has slept, and how tired they feel upon waking (Angelhoff et al., 2017). Parental sleep when their child is sick was referred to as experiencing poor sleep quality that included descriptions such as waking earlier (Mihaila & Hartley, 2016), frequent night awakenings (Yuwen et al., 2016), and less sleep (Mihaila & Hartley, 2016; Yuwen et al., 2016). Parents being supported has been shown to impact sleep quality. When parents feel supported, it has been found they perceive their sleep quality as good, despite reporting more nocturnal awakenings (Angelhoff et al., 2017).

##### Prolonged sleep latency

Sleep latency, the time taken to fall asleep (McLoone et al., 2013; Shaki et al., 2011), was described as prolonged in parents caring for sick children. Larger proportions of parents with sick children reported trouble falling asleep (Wright, 2010) and taking longer to fall asleep (Keilty et al., 2018; Lee & Kimble, 2009; Mcbean & Schlosnagle, 2016; Wright, 2010; Wright et al., 2006) than healthy/typically developing children parents. For example, Keilty et al. (2018) found twice as many parents of children dependent on medical technology took more than 30 min to fall asleep than controls.

##### Frequent sleep disturbance

The continuity of sleep for parents with sick children was described as disturbed (Angelhoff et al., 2015; Chu & Richdale, 2009; Feeley et al., 2014; Gedaly‐Duff et al., 2006; Goldman et al., 2012; Johnson et al., 2018; Meltzer & Pugliese, 2017; Stremler et al., 2011; Wayte et al., 2012). Various terms were used to describe waking during the night including disturbance (e.g., Ortiz‐Rubio et al., 2021), disruption (e.g., Keilty et al., 2018), interruption (e.g., Chu & Richdale, 2009), fragmented (e.g., Feeley et al., 2019), nocturnal awakenings (e.g., Angelhoff, Edéll‐Gustafsson, et al., 2019), arousal (e.g., Stremler et al., 2014), wake after sleep onset (e.g., Yuwen et al., 2016) (see Appendix S3, Table of references, point 3). Variability was found in what was meant by sleep disturbance. Often described as entailing disturbance after falling asleep, such as more interruptions (Cottrell & Khan, 2005), it was also defined as a few hours of sleep (Cottrell & Khan, 2005) and taking longer than 30 min to fall asleep (Gedaly‐Duff et al., 2006).

Disturbed sleep was found to involve many factors such as waking up several times after falling asleep due to worries or troubling thoughts (Angelhoff et al., 2015; Cheezum et al., 2013), stress (Edell‐Gustafsson et al., 2014) or anxiety (Feeley et al., 2019) related to the child's condition, providing care for the child (Cheezum et al., 2013; Meltzer & Mindell, 2006), changes to sleep arrangements (Heaton et al., 2006) and patterns (Heaton et al., 2006; Ortiz‐Rubio et al., 2021), checking on their child and sleeping lightly (Cheezum et al., 2013), and co‐sleeping with their child (Adiga et al., 2014). When explored, gender differences were also noted, with mothers having more sleep disturbance than fathers (Al Maghaireh et al., 2017). The context of the hospital was often investigated with parents sleeping with their child (Franck et al., 2014; Stremler et al., 2014) with the following highlighted as disturbing parents' sleep: noise (e.g., Nassery & Landgren, 2018), light (e.g., Stickland et al., 2016), sleeping accommodation (e.g., Al Maghaireh et al., 2017; McLoone et al., 2013), treatment or medications (Feeley et al., 2014), interruptions from healthcare professionals (e.g., McLoone et al., 2013) and child awakenings (Feeley et al., 2014), as well as being in an unfamiliar environment (Stremler et al., 2011) (see Appendix S3, Table of references, point 4). The home context was also reviewed. It was found that parents slept better at home compared with the hospital (Bevan et al., 2019; Meltzer et al., 2012) yet could still experience disturbances such as noise from equipment (Heaton et al., 2006).

##### Shorter sleep duration

Optimal sleep was often described as 7–9 h per night (Herbert et al., 2014; Mörelius & Hemmingsson, 2013). Parents caring for a sick child were found to sleep less than 7 h per day (e.g., Jaser et al., 2017) (see Appendix S3, Table of references, point 5). Sleep duration (e.g., Larson et al., 2012) was a term often used along with lack of sleep (e.g., Heaton et al., 2006), sleep loss (e.g., Bevan et al., 2019), sleep less (e.g., Wright et al., 2006), sleep quantity (e.g., Cheezum et al., 2013), sleep efficiency (e.g., Boergers et al., 2007), and total sleep time (e.g., Goldman et al., 2012) (see Appendix S3, Table of references, point 6). Similarly, parents of sick children were described as having inadequate (Lee & Hsu, 2012; Wright et al., 2006; Yuwen et al., 2016), insufficient (Byars et al., 2020; Moore et al., 2006), deficient (Meltzer & Booster, 2016; Meltzer et al., 2015), or restricted sleep (Stremler et al., 2014). Earlier wake times were noted, for example, in parents of children with autism spectrum disorders (Meltzer, 2008) and ventilator dependency (Meltzer & Mindell, 2006) when at home. Later wake times were also found for parents rooming in with their children at hospital in comparison with home due to more awakenings (Meltzer et al., 2012). Some studies reported that parents caring for sick children had difficulties falling back to sleep once awoken, with others falling straight back to sleep (Angelhoff et al., 2015). Some parents did not sleep if they were not near their child (Stremler et al., 2014). These studies highlight that sleep is an individual experience that can be influenced by a range of factors, including worry (Franck et al., 2014; Herbert et al., 2014), stress (Gallagher et al., 2009; Neu et al., 2014), coping strategies (Al Maghaireh et al., 2017; Cadart et al., 2018), support (Angelhoff, Askenteg, et al., 2019; Angelhoff et al., 2015; Gallagher et al., 2009), and acceptance of sleep loss by the parents (Angelhoff, Askenteg, et al., 2019).

In summary, despite the various research around the concept of parental sleep when their child is sick, the concept has not been defined or used explicitly in the scientific literature. The implied meaning and variability of terms used adds to the lack of clarity.

#### Pragmatic

The pragmatic principle considers the usefulness of the concept in terms of how it explains or describes phenomena in a discipline (Ruel & Motyka, 2009) and whether it has been operationalised. Most of the studies reviewed described the applicability and usefulness of parental sleep when the child is sick. The approaches to parental sleep when their child is sick will now be reviewed.

##### Subjective measurements

Most often, the use of this concept was related to subjective measurements of parental sleep when their child is sick. The most frequently used tool in this dataset was the Pittsburgh Sleep Quality Index (PSQI) used in 34 of the 74 research studies (e.g., Lopez‐Wagner et al., 2008) and comes validated in many languages (Liu et al., 2021; Nozoe et al., 2016; Ridolo et al., 2015; Shaki et al., 2011) (see Appendix S3, Table of references, point 7). The PSQI assesses sleep quality and disturbances over the past month and measures sleep in seven domains on a four‐point Likert scale on subjective sleep quality, sleep latency, sleep duration, habitual sleep efficiency, sleep disturbances, use of medications, and daytime dysfunction. The component scores are summed to produce a global score (range 0–21) (Adiga et al., 2014). A PSQI global score of ≥5 is suggestive of significant sleep disturbance (Adiga et al., 2014; Chu & Richdale, 2009). The tool has been used frequently in concert with other tools to produce correlations with depression (e.g., Filiz et al., 2020), anxiety (e.g., Chu & Richdale, 2009), stress (e.g., Gallagher et al., 2009), sleepiness (e.g., Cadart et al., 2018), parental adjustment/impact to their child's condition (e.g., Herbert et al., 2014), burden (e.g., Yilmaz & Alemdar, 2020), quality of life (e.g., Tietze et al., 2014), worry (Herbert et al., 2014), fatigue (e.g., Larson et al., 2012), memory (Mcbean & Schlosnagle, 2016), pain (Albayrak et al., 2019), and support (Gallagher et al., 2009) (see Appendix S3, Table of references, point 8). The PSQI has also been used with actigraphy (Feeley et al., 2021; Keilty et al., 2018; Meltzer, 2008) and sleep diary data (Feeley et al., 2021; Keilty et al., 2018; Meltzer & Moore, 2008). Despite its use, limitations of the tool have been noted as it excludes situational specific sleep disruptions with some authors including additional questions to explore reasons for parents' sleep disturbance (Herbert et al., 2014; Meltzer & Booster, 2016; Meltzer & Mindell, 2006). Other tools used to measure parental sleep when their child is sick in the literature included the Epworth Sleepiness Scale (Cadart et al., 2018; Cheezum et al., 2013; Goldman et al., 2012; Keilty et al., 2018; Tietze et al., 2014; Yilmaz & Alemdar, 2020), the General Sleep Disturbance Scale (Lee & Hsu, 2012; Lee & Kimble, 2009; Lee et al., 2007; Zupanec et al., 2010), the 24‐Hour Sleep Patterns Inventory (Meltzer et al., 2010; Meltzer & Mindell, 2006). Less frequently used were the Parent's Sleep Habits Questionnaire (Boergers et al., 2007), Uppsala Sleep Inventory (Angelhoff et al., 2017), St Mary's Hospital Sleep Questionnaire (McLoone et al., 2013), the Patient‐Reported Outcomes Measurement Information System (PROMIS) Sleep Disturbance Form (Feeley et al., 2021; Meltzer & Pugliese, 2017), to name a few. A small number of studies did not use validated tools (Bourke‐Taylor et al., 2013; Chamlin et al., 2005; Johnson et al., 2018). One study reviewed the Caregiver Burden Inventory as a possible screening tool for sleep disturbance but required further validation with longitudinal studies and to be assessed with other child chronic conditions (Ortiz‐Rubio et al., 2021).

##### Subjective explorations

Many studies used qualitative approaches to explore the concept. Interviews with parents regarding their sleep experiences were conducted (Angelhoff, Askenteg, et al., 2019; Angelhoff et al., 2015, 2020; Edell‐Gustafsson et al., 2014; Nassery & Landgren, 2018; Neu et al., 2014; Stickland et al., 2016), and less common in combination with diaries and photos (Heaton et al., 2006), and questionnaires (Cheezum et al., 2013). Open questions in the form of a qualitative questionnaire format varied from being used solely (Feeley et al., 2019; Stremler et al., 2011), including closed questions (Wright, 2010; Wright et al., 2006), and alongside actigraphy (Franck et al., 2014) and other questionnaires (McCann, 2008; Zupanec et al., 2010). Similar to the limitations noted with the PSQI, qualitative interviews were used alongside the Epworth Sleepiness Scale to capture sleep problems faced by parents (Cheezum et al., 2013).

Appropriateness was at times questioned. For example, some studies used a lot of questions (Cheezum et al., 2013), did not describe the analysis process or method (Cheezum et al., 2013; Stickland et al., 2016), or used closed questions (Nassery & Landgren, 2018) for qualitative studies. Whereas other studies reported the credibility of the analysis (Heaton et al., 2006; Stremler et al., 2011; Wright, 2010) or justified why a particular method was used (Angelhoff, Askenteg, et al., 2019; Angelhoff et al., 2015) thus establishing trustworthiness.

##### Objective measurements

Wrist actigraphy was the most used objective measure used in 13 articles (e.g., Bevan et al., 2019) (see Appendix S3, Table of references, point 9). It was noted to be highly reliable and validated to differentiate sleep from wake with some studies using sleep diaries to aid interpretation of the data (Keilty et al., 2018; Lee & Hsu, 2012; Lee & Kimble, 2009; Lee et al., 2007; Meltzer, 2008; Stremler et al., 2014; Yuwen et al., 2016). Actigraphy was also often recommended for future studies to remove biased reporting (e.g., Mihaila & Hartley, 2016) and over estimations (McLoone et al., 2013) from research using self‐reported tools or to complement subjective measures (Angelhoff et al., 2017) (see Appendix S3, Table of references, point 9). Although not researched in the literature of this concept analysis, polysomnography was also recommended (Larson et al., 2012; Lee & Hsu, 2012; Mcbean & Schlosnagle, 2016; Meltzer & Pugliese, 2017; Moore et al., 2006; Yang et al., 2020; Zupanec et al., 2010).

##### Study participants

Of the studies that included quantitative methods, 34 of the 56 included fewer than 100 participants (e.g., Feeley et al., 2021) and only eight studies reported power calculations (e.g., Ortiz‐Rubio et al., 2021) (see Appendix S3, Table of references, point 10). In all approaches to the concept whether qualitative, quantitative, or mixed‐methods, more mothers took part in the research than fathers (see Appendix S2, Literature overview), with some studies excluding fathers entirely as it was stated that generally, mothers were the primary caregiver (Neu et al., 2014) or insufficient results from the fathers for reliable statistical analysis were received (Yılmaz et al., 2008). Many studies highlighted the need to conduct more studies with fathers (Meltzer, 2008; Meltzer & Booster, 2016; Meltzer et al., 2010; Mörelius & Hemmingsson, 2013; Yılmaz et al., 2008), especially considering they are becoming increasingly involved in the care of their children (Mörelius & Hemmingsson, 2013). Further research on single parents was also recommended to gain a complete view (Angelhoff et al., 2015). Conducting longitudinal research was another recommendation (e.g., Albayrak et al., 2019) (see Appendix S3, Table of references, point 11).

In summary, the concept is recognisable in clinical practice yet still in its early stages. It is likely that no single approach currently captures the whole experience. The use of quantitative tools is significant and shows an application of parental sleep when their child is sick as a concept of interest to practice. The difficulty remains in the usefulness of these tools and the samples used.

#### Linguistic

The consistent and appropriate use of the concept was explored along with the context fit through the linguistic principle.

##### Variety of terms

Due to not having a specific definition, the analysis of the linguistic principle revealed that a variety of terms were used in the literature to describe parental sleep when their child is sick. Across disciplines, there was some consistency in use and the meaning of terms regardless of the population, and the contextual setting used to explore the concept. Parental sleep when their child is sick was commonly described in the epistemological principle by sleep quality, sleep disturbance, sleep patterns, sleep duration, sleep efficiency, sleep latency, to name a few. As mentioned in the pragmatic principle, quantitative research dominates the research base, with the PSQI and actigraphy being common tools used and employing many of these terms. Yet, these terms were also present in the qualitative data suggesting that implied meaning is to some extent consistent.

##### Overlapping descriptions

The inconsistency occurs with the overlap of some of these terms described in the epistemological principle. For example, poor sleep quality descriptions included waking earlier (Mihaila & Hartley, 2016) and less sleep (Mihaila & Hartley, 2016; Yuwen et al., 2016) (also used in the description of shorter sleep duration), and frequent night awakenings (Yuwen et al., 2016) (also used in frequent sleep disturbance descriptions). Similarly, frequent sleep disturbance was also defined as a few hours of sleep (Cottrell & Khan, 2005) (also used in shorter sleep duration term*)* and taking longer than 30 min to fall asleep (Gedaly‐Duff et al., 2006) (also used in prolonged sleep latency term*)*.

##### Interchangeable terms

Similar terms were often used interchangeably, as noted in the epistemological principle. For example, the Bourke‐Taylor et al. (2013) study title includes sleep disruption but throughout the article, sleep interruption was more dominantly used. Often in the literature, it is not known whether similar terms have the same meaning or mean something entirely different, and it is often left to the reader to interpret. Lack of terminology clarity and consistency can make it difficult to interpret and apply the findings.

The current tools available such as the PSQI, may also contribute to the inconsistency of terms. For example, the scoring of the PSQI global score of 5 or greater had various terms used, including the score indicating poor sleep quality (Wayte et al., 2012), significant sleep disturbance (Adiga et al., 2014), poor sleep (Chu & Richdale, 2009), clinically disturbed sleep (Feeley et al., 2014), a marker of sleep disturbance in insomnia (Gallagher et al., 2009) or as identifying a sleep disorder (Hansen et al., 2012), highlighting no consensus. Yılmaz et al. (2008) reported a different scale in which 0–5 indicated good sleep, 6–10 bad sleep, and scores above 10 indicated chronic sleep disorder. Sleep disorder was described in the literature as prolonged sleep latency, and shortened sleep duration (Tietze et al., 2014) and insomnia was described as difficulty initiating and maintaining sleep (Meltzer & Booster, 2016) which were very similar to the terms mentioned earlier, adding to the inconsistency.

In summary, the implied meaning of the concept, parental sleep when their child is sick, within the literature is often inconsistent and has the potential to be interpreted differently. The variety of synonyms used and the lack of conceptual precision allows wide variation in attributed features providing a need for clarification.

#### Logical

The logical principle assesses whether the concept holds its boundaries when integrated with other related concepts (Penrod & Hupcey, 2005). This principle explores the theoretical integration of the concept “parental sleep when their child is sick” with related concepts.

##### Interrelated concepts

Psychological factors, for example, mood (Angelhoff et al., 2017), depression (Cheezum et al., 2013; Chu & Richdale, 2009; Filiz et al., 2020; Liu et al., 2021; Yang et al., 2020), anxiety (Al Maghaireh et al., 2017; Yang et al., 2020), stress (Al Maghaireh et al., 2017; Bevan et al., 2019; Bourke‐Taylor et al., 2013; Feeley et al., 2021), fatigue (Gedaly‐Duff et al., 2006; Larson et al., 2012), exhaustion (Mörelius & Hemmingsson, 2013; Ramirez et al., 2019) were often researched alongside parental sleep when their child is sick. The literature shows that the boundaries between parental sleep when their child is sick and these factors are often blurred. Noted as a controversial topic, the direction of the relationship between sleep loss and other concepts is often unclear. Yılmaz et al. (2008) found that sleep and anxiety‐depression parameters influence each other and suggested that a two‐sided influence exists. Similarly, McLoone et al. (2013) did not clarify the direction of the relationship between anxiety and poor sleep. Adiga et al. (2014) could also not provide information regarding the cause and effect relationship between parents and bedsharing with their child. Chu and Richdale (2009) argued that inferences of direction are difficult as various factors can interrelate adding to the lack of boundaries within this concept. Similarly, cross‐sectional study designs make it impossible to establish causal relationships, for example, between caregiver burden and sleep quality (Ortiz‐Rubio et al., 2021) and sleep and asthma (Meltzer & Pugliese, 2017).

##### Differentiation

Differentiation was shown in comparisons with parents of typically developing children. For example, Shaki et al. (2011) found mothers of children with epilepsy had a seven‐fold occurrence of sleep disturbances when compared with mothers of non‐epileptic children. Similarly, unlike parents of typically developing children whose sleep often improves over time as the child ages, parental sleep when their child is sick involves long‐term care requirements and sleep disturbance leading to detrimental effects on parents' health (Bourke‐Taylor et al., 2013). In contrast, boundaries were not maintained with parents and the child's condition. It was found that the child's condition impacted parents' sleep (Byars et al., 2020; Cadart et al., 2018; Feeley et al., 2021; Nassery & Landgren, 2018), with some arguing a reciprocal relationship (Boergers et al., 2007) where poor sleep hygiene practices implemented by parents also have a role to play and impact the child's sleep (Boergers et al., 2007; Zupanec et al., 2010).

##### Theoretical integration

Given that the concept has not been explicitly defined, attempts at theoretical integration were limited. Only 10 studies discussed theoretically parental sleep when their child is sick (Angelhoff et al., 2015; Feeley et al., 2021; Feeley et al., 2014; Lee & Hsu, 2012; McCann, 2008; Meltzer & Moore, 2008; Mihaila & Hartley, 2016; Nassery & Landgren, 2018; Stremler et al., 2014; Stremler et al., 2011). The family‐centred care model was often mentioned in the literature (Liu et al., 2021; McCann, 2008; Meltzer & Moore, 2008; Nassery & Landgren, 2018; Pouraboli et al., 2019; Stremler et al., 2011, 2014) and noted as encouraging collaboration and partnership between families when making healthcare decisions and considering the family as a unit of care (Nassery & Landgren, 2018). McCann (2008) suggests that nurses need to take greater responsibility for ensuring that parents obtain minimum sleep when staying in hospital yet do not advise what minimum sleep is. Meltzer (2008) states that medical communities need to consider that parents may experience sleep disruptions related to their child when at home. Indirect references to parental sleep when their child is sick were made in additional theoretical applications, including the biopsychosocial model (Cottrell & Khan, 2005; Larson et al., 2012; Yılmaz et al., 2008), Cognitive Activation Theory (Mörelius & Hemmingsson, 2013), Robinson's Model of Family Perspective (Gedaly‐Duff et al., 2006), and the Social‐Ecological Framework (Meltzer & Booster, 2016).

More applied applications included Karasek, Theorell's (Karasek & Theorell, 1990) demand–control–support model (Angelhoff et al., 2015), Lazarus and Folkman's (1984) theory of stress, appraisal, and coping (Feeley et al., 2014, 2021), and the sleep–stress cycle (Mihaila & Hartley, 2016) emphasising the stress–sleep association. Angelhoff et al. (2015) adapted the demand–control–support model of work‐related stress to sleep and suggested that low perceived control creates stress responses that may lead to psychological or physical health problems such as sleep disruption. The model also addresses the impact of support in facilitating sleep revealing various factors that contribute or impact sleep highlighting an interaction of factors. Similarly, Feeley et al. (2014) adapted Lazarus and Folkman (1984) theory of stress, appraisal, and coping to parental sleep and highlighted that sleep quality and caregiver burden have been shown to influence stress in maternal caregivers and may act as antecedents to the maternal caregiver's appraisal of the situation (Fletcher et al., 2008; Gallagher et al., 2009). Feeley et al. (2021) used the theory as a guiding framework for their study. Mihaila and Hartley (2016) discussed the mutual impact of stress and sleep in their cycle on the child's behaviour and the psychological well‐being of the parent. This model also covers the quantity of total sleep time and, interestingly, covers the perceived quality of sleep in maintaining health‐related well‐being (Lee, 2003; Lee & Hsu, 2012). Lee's (Lee, 2003) impaired sleep model described in the study of Lee and Hsu (2012), is more applied to the concept and suggests that sleep deprivation and sleep disruption are characterised by fragmented sleep during the night and contribute to physical and mental outcomes.

In summary, the conceptual boundaries of parental sleep when their child is sick were clear with some related concepts (parents of typically developing children) and blurred with others (e.g., stress, depression, mood, anxiety, fatigue, exhaustion, child's condition). The concept has been applied within theoretical applications either indirectly or to some extent directly, yet there was not a specific theory/model/framework to parental sleep when their child is sick. The concept often does not maintain its identity as its boundaries are often blurred and were not indistinguishable within theoretical applications.

The evidence reviewed supports the utility of the concept “parental sleep when their child is sick” (pragmatic principle). However, there is a lack of a precise definition of the concept with a reliance on implied meaning (epistemological principle), which has led to the use of the concept being inconsistent (linguistic principle), and frequently blurred when theoretically positioned with other concepts (logical principle).

#### Phase 3; stage 3: conceptual components

**FIGURE 2:**
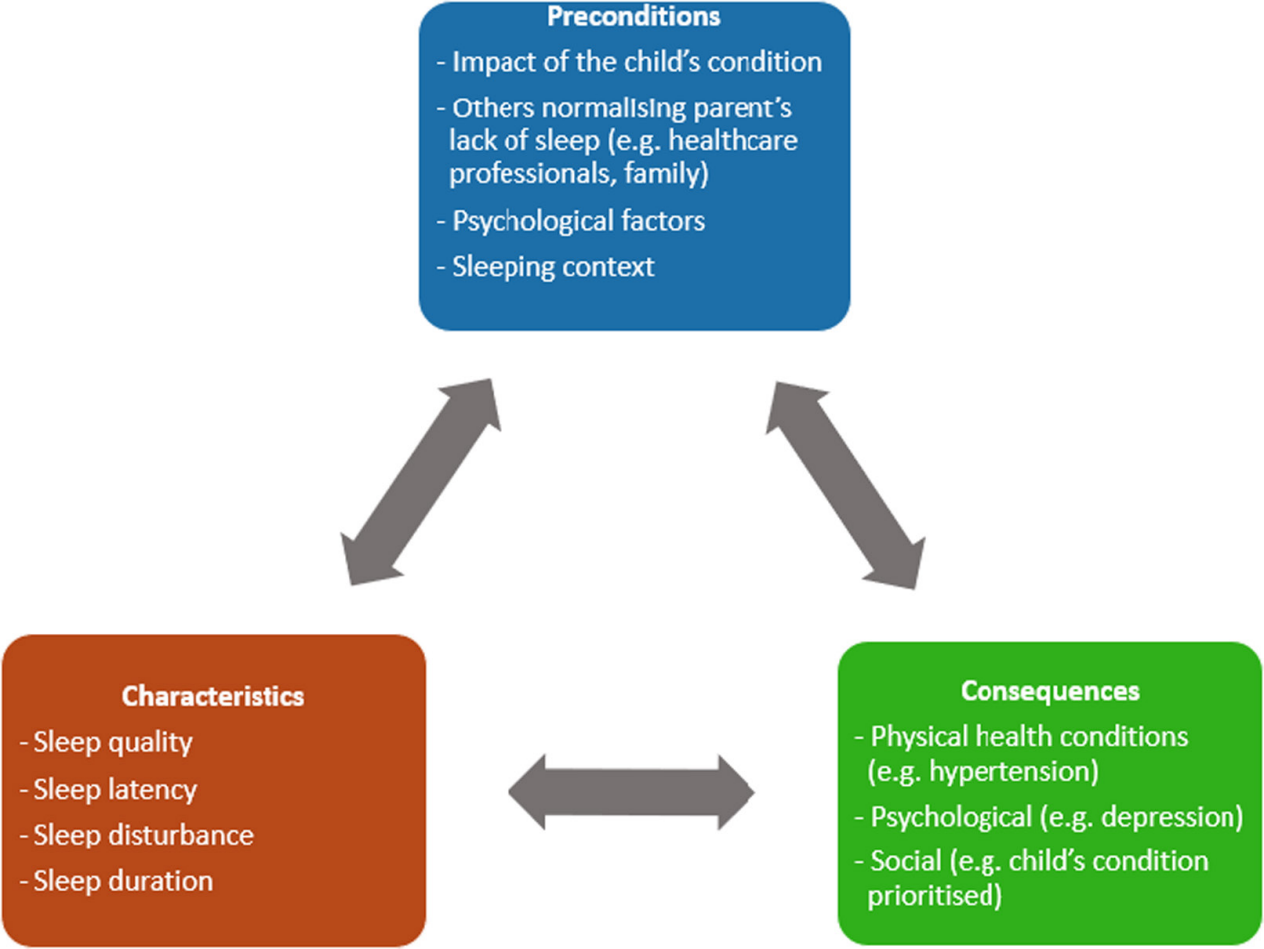
Conceptual components of parental sleep when their child is sick

Informed by the findings from each principle and additional notes and coding made to the three aspects of the conceptual components, the conceptual components of parental sleep when their child is sick are now organised into preconditions (phenomena/events that precede an instance and that influences the concept), characteristics (frequent words or expressions used to describe the experience of the concept), and outcomes (the consequences that follow the occurrence of the concept) (Waldon, 2018). These conceptual insights assist in advancing clarity and understanding of the concept and contribute to the theoretical definition, the final product of the principle‐based concept analysis. Figure 2 shows the conceptual model of parental sleep when their child is sick and the interactions between the components.

#### Preconditions

The child's condition was the primary precondition which has been shown to involve parents adjusting their sleep patterns, attending to their child more during the night, and may result in parents accepting their sleep loss (Angelhoff, Askenteg, et al., 2019; Heaton et al., 2006). The impact can further be heightened by family, friends, and healthcare professionals normalising the lack of sleep for parents and not seeking help (Angelhoff, Askenteg, et al., 2019). Other features linked to the child's condition include psychological factors including stress, anxiety, worry, for example, in obtaining a diagnosis. Context, in terms of the sleeping environment such as staying with their hospitalised child, can influence parents' sleep, the accommodation provided, and whether parents have their own room or share the room with others (Stremler et al., 2011).

#### Characteristics

Factors suggested as characteristics of parental sleep when their child is sick included poor sleep quality, prolonged sleep latency, frequent sleep disturbance, and shorter sleep duration. What coping strategies and support parents have in place also influences the concept. For example, coping was linked to parents practising yoga (Neu et al., 2014), meditation (Neu et al., 2014; Stremler et al., 2011) and exercise (Angelhoff et al., 2015), spirituality and faith (Stremler et al., 2011) and found to help parents to practise better sleep. Support included the family, healthcare professionals/nursing support/home support/care plans (Angelhoff et al., 2015), information support on the condition (Cheezum et al., 2013; Feeley et al., 2014; Larson et al., 2012; McCann, 2008), and sleep hygiene to improve sleep (Cottrell & Khan, 2005; McLoone et al., 2013; Shaki et al., 2011; Stremler et al., 2014; Yuwen et al., 2016; Zupanec et al., 2010).

#### Outcomes

The consequences of sleep are focused on biopsychosocial factors. Biologically, cardiovascular disease (Angelhoff, Askenteg, et al., 2019; Cheezum et al., 2013; Johnson et al., 2018; Ramirez et al., 2019), hypertension (Johnson et al., 2018; Nassery & Landgren, 2018), respiratory (Cheezum et al., 2013), type 2 diabetes (Angelhoff, Askenteg, et al., 2019; Cheezum et al., 2013; Nassery & Landgren, 2018), obesity (Angelhoff, Askenteg, et al., 2019; Cheezum et al., 2013; Feeley et al., 2019; Nassery & Landgren, 2018; Ramirez et al., 2019) were highlighted as potential risks from sleep loss. Psychologically, sleep loss was linked to mood (Angelhoff et al., 2015; Angelhoff, Askenteg, et al., 2019), anxiety (Al Maghaireh et al., 2017; Cadart et al., 2018), depression (Al Maghaireh et al., 2017; Cadart et al., 2018; Filiz et al., 2020; Liu et al., 2021; Meltzer & Pugliese, 2017), fatigue (Meltzer & Mindell, 2006), concentration (Angelhoff, Askenteg, et al., 2019; Angelhoff et al., 2017, 2020; Gallagher et al., 2009), memory (Mcbean & Schlosnagle, 2016) or impaired cognitive function (Angelhoff, Askenteg, et al., 2019), stress (Angelhoff et al., 2015; Bevan et al., 2019; Feeley et al., 2021), lack of self‐care (Stremler et al., 2011), exhaustion (Ramirez et al., 2019), and daytime function such as difficulties to remain awake and/or maintaining enthusiasm with tasks (Herbert et al., 2014; Wright, 2010). In particular, decision‐making on the child's care was noted to be impacted by a lack of sleep (Heaton et al., 2006; Herbert et al., 2014; Johnson et al., 2018; McLoone et al., 2013). Socially, the child's health was often prioritised, and/or parents experienced a lack of energy to engage in activities and social events (Angelhoff, Askenteg, et al., 2019). Parents were reported to miss their social life with friends and relatives (Edell‐Gustafsson et al., 2014). Sleep disruption was associated with tiredness and limiting the parents' ability to work and socialise (Heaton et al., 2006). Social support was also found to be associated with longer sleep (Johnson et al., 2018).

#### Phase 3; stage 4: theoretical definition

The theoretical definition is presented based on the findings of the principle‐based concept analysis, including implied meanings and the conceptual components. The following theoretical definition is suggested:

Parental sleep when their child is sick is an individualised experience characterised by sleep quality based on sleep latency, sleep disturbances, and sleep duration and is influenced by the child's condition, parents' sleeping environment, coping strategies, health problems, support systems, and interventions which can shape parent's biopsychosocial health outcomes.

## 4 DISCUSSION

The purpose of this concept analysis on parental sleep when their child is sick was to reveal the current state of the empirical knowledge surrounding the concept to facilitate its advancement. The identified conceptual components and the interrelationships between them have been integrated into a recommended definition of parental sleep when their child is sick. In addition, the analysis provides further points for consideration to better understand and utilise this concept.

Despite the utility of the concept and the recognition of the importance of parental sleep, a conceptually derived definition of the concept was not present. Parental sleep when their child is sick contrasted to adequate sleep definitions (Lee & Hsu, 2012; Lee & Kimble, 2009). With no explicit definition to describe the concept (epistemological), the validity of the tools designed to measure the phenomenon are vulnerable with regard to this concept (pragmatic), with the interpretation of the implicit meanings inconsistent (linguistic), and the concept's identity often blurred when applied theoretically (logical) with other related concepts. Gerring (2001) suggests having homonymy (multiple meanings for the same term) and synonymy (different terms with the same, overlapping meanings) are problematic. For example, difficulties arise when searching the empirical literature (Proctor et al., 2013), possibly resulting in important literature being missed. The use of multiple terms poses barriers to communication and progress for applying research findings (McKibbon et al., 2010).

Some authors have suggested that developing a theory would render the concept more useful (Løyland et al., 2019). Our conceptual model of parental sleep when their child is sick addresses this by integrating and explaining the mutual relations of the underlying/related concepts.

### 4.1 Strengths and limitations

The strengths of this analysis included each article being read twice, and a pilot test with a selection of articles was undertaken with three researchers aiding reliability and validity of the analysis. Consultation of the findings was reviewed by a varied research team. Only English language articles were included, which may potentially limit the findings. A small number of studies included did not use validated tools (Bourke‐Taylor et al., 2013; Byars et al., 2020; Chamlin et al., 2005; Johnson et al., 2018; Ramirez et al., 2019) and have therefore not undergone psychometric testing for reliability, validity, and sensitivity which questions the accuracy of the reported data.

### 4.2 Further development of the concept

Research gaps included interventions to promote parental sleep such as support/information provision (e.g., Pouraboli et al., 2019), assessing parents’ sleep (e.g., Feeley et al., 2021), addressing under researched areas such as fathers (e.g., Yilmaz & Alemdar, 2020), single parent sleep (Angelhoff et al., 2015), longitudinal research (e.g., Albayrak et al., 2019), the need for larger samples in quantitative research (e.g., Filiz et al., 2020), and randomised controlled trials (Ullas et al., 2021) (see Appendix S3, Table of references, point 11). The findings from this study highlight the need to develop a tool for parental sleep when their child is sick.

## 5 CONCLUSIONS

This phased principle‐based concept analysis provided a rigorous evaluation of parental sleep when their child is sick using a triangulation of methods. The results of this analysis have shown many gaps in our understanding of this concept, resulting in a lack of clarity, consistency, and definition. This concept analysis is a starting point for concept advancement. The results of this study have pragmatic implications for scientific inquiry and clinical practice. How we define parental sleep when a child is sick impacts how it is studied and how interventions are planned. This concept analysis and theoretical definition serve as a basis for future research, particularly in further clarity of this concept.

## CONFLICT OF INTEREST

The authors declare that they have no conflict of interest.

## AUTHOR CONTRIBUTIONS

SS, EM: Involved in the conception and design of the study; SS: Conducted the searches and acquired data; SS, MT, EM: Screened articles; SS, JS, CA: Adapted and pilot tested the quality criteria assessment; SS: Conducted the qualitative analysis and interpretation of data; EM: Oversaw the analysis and interpretation of the data; SS: Drafted the manuscript; SS, MT, JS, CA, EM: Revised the manuscript critically for important intellectual content; SS, MT, JS, CA, EM: Gave final approval of the version to be published.

## Supporting information

App S1‐S3

Figure 1

## Data Availability

All data generated or analysed during this study are included in this published article [and its supplementary information files].
