# Supplementary material for: Parental sleep when their child is sick: A phased principle‐based concept analysis
Source: J Sleep Res. 2022 Apr 25;31(5):e13575. doi: 10.1111/jsr.13575 (PMC9786861; doi:10.1111/jsr.13575)
Supplement: Supplementary file 1 — App S1‐S3 [file JSR-31-e13575-s001.docx]

**Supplementary 1:** Quality criteria for a Phased Principle-Based Concept Analysis adapted to ‘parental sleep when their child is sick’

| **Epistemology** | **Is the concept *‘parental sleep when their child is sick’* clearly defined and differentiated from other concepts?**  This principle critically explores the clarity of a definition for the concept*.* It focuses on the discipline’s distinction of a concept within the knowledge base. It involves determining if the principle is clearly defined and differentiated from other concepts. |
| --- | --- |
|  | **E1:** Is *‘parental sleep when their child is sick’* defined?  **When to apply:**  **Yes:** An understandable and detailed definition of *‘parental sleep when their child is sick’* is clear in the literature.  **Partly:** Implied conceptual meaning of *‘parental sleep when their child is sick’* is offered through a sentence (either from the findings or from cited research) or using attributes and characteristics (frequent words or expressions used to describe the experience of *‘parental sleep when their child is sick’*).  **No:** There is not a clear and detailed definition or implied meaning of *‘parental sleep when their child is sick’* in the literature.  **E2:** Is *‘parental sleep when their child is sick’* differentiated/distinguished from other concepts (e.g., *child’s sleep, controls, sleep diseases, exhaustion, depression, mood, and other biopsychosocial health*)?  **When to apply:**  **Yes:** There is clearly documented differentiation/distinction between *‘parental sleep when their child is sick’* and other key concepts *(e.g., child’s sleep, controls, sleep diseases, exhaustion, depression, mood, and other biopsychosocial health).*  **Partly:** Other key concepts *(e.g., child’s sleep, controls, sleep diseases, exhaustion, depression, mood, and other biopsychosocial health)* are presented as uniquely different yet related (e.g., as antecedents/preconditions, consequences, or outcomes) to *‘parental sleep when their child is sick’* and at the same time there may also be some distinction shown with other concepts.  **No:** Other key concepts *(e.g., child’s sleep, controls, sleep diseases, exhaustion, depression, mood, and other biopsychosocial health)* are not /distinguished from *‘parental sleep when their child is sick’.* |
| **Pragmatic** | **Is the concept *‘parental sleep when their child is sick’* applicable and useful?**  This principle explores the applicability and usefulness of the concept and to what degree has it been operationalised (e.g., ethical considerations, sample used, measures used, policies/interventions developed). The data are analysed from the perspective of usefulness. |
|  | **P1:** Is *‘parental sleep when their child is sick’* useful and applicable (e.g., researching *‘parental sleep when their child is sick’* and/or applies to *‘parental sleep when their child is sick’* through the study purpose/aims of the research/identification of knowledge gaps, findings, and recommendations) within health disciplines (e.g., beneficial to healthcare, clinical practice, or research)?  **When to apply:**  **Yes:** It is clearly stated how *‘parental sleep when their child is sick’* is useful and applicable for describing or explaining phenomena within the health discipline (e.g., beneficial to healthcare, clinical practice, or research).  **Partly:** It is clearly stated to some extent how *‘parental sleep when their child is sick’* is useful and applicable for describing or explaining phenomena within the health discipline (e.g., beneficial to healthcare, clinical practice, or research).  **No:** It is not clearly stated how *‘parental sleep when their child sick’* is useful and applicable for describing or explaining phenomena within the health discipline (e.g., beneficial to healthcare, clinical practice, or research).  **P2:** Has the concept *‘parental sleep when their child is sick’* been appropriately measured/explored and evaluated (e.g., ethical considerations, sample, measures used, policies/interventions developed)?  **When to apply:**  **Yes:** *‘Parental sleep when their child is sick’* has been measured/explored and evaluated as appropriate to improve health outcomes.  **Partly:** *‘Parental sleep when their child is sick’* has been measured/explored and evaluated to some extent as appropriate to improve health outcomes.  **No:** *‘Parental sleep when their child is sick’* has not been measured/explored and evaluated appropriate to improve health outcomes. |
| **Linguistic** | **Is the concept *‘parental sleep when their child is sick’* used consistently and appropriately within the scientific literature?**  This principle is concerned with the consistency in use and meaning, as well as the fit of the concept in the context of use across a variety of contexts. This principle evaluates the appropriate use of the concept. In this assessment, consistency in use and meaning are considered. The context in which the concept has use and meaning is also considered. |
|  | **L1:** Is *‘parental sleep when their child is sick’* or the language, or the key attributes and characteristics around *‘parental sleep when their child is sick’* identified and used **consistently** within the whole article?  **When to apply the code:**  **Yes:** *‘Parental sleep when their child is sick’* is used consistently or the language or the key attributes and characteristics that describe *‘parental sleep when their child is sick’* are used consistently within the article (e.g., *‘parental sleep when their child is sick’* is used or key attributes/characteristics are used consistently throughout the article).  **Partly:** *‘Parental sleep when their child is sick’* to some extent is used consistently or the language or the key attributes and characteristics to describe *‘parental sleep when their child is sick’* are to some extent used consistently within the article (e.g., other terms are used but key terms are identifiable and to some extent used consistently).  **No:** *‘Parental sleep when their child is sick’* is not used consistently or the language or the key attributes and characteristics to describe *‘parental sleep when their child is sick’* are not used consistently within the article (e.g., various terms are used).  **L2:** Is *‘Concept’* or the language or the key attributes and characteristics around *‘parental sleep when their child is sick’* used **appropriately** within the context of the article?  **When to apply the code:**  **Yes:** *‘Concept*’ is described appropriately or the key language or the attributes and characteristics to describe *‘Concept’* are described appropriately.  **Partly:** *‘Concept’* to some extent is described appropriately or the language or the key attributes and characteristics to describe *‘Concept’* are to some extent described appropriately.  **No:** *‘Concept’* is not described appropriately or the language or the key attributes and characteristics to describe *‘Concept’* are not described appropriately. |
| **Logical** | **Does the concept ‘*parental sleep when their child is sick’*** **hold its boundaries when integrated with other concepts?**  This principle refers to the integration of the concept with related concepts. The data are analysed to determine if the concept becomes indistinct when positioned theoretically with other concepts. Ideally, a concept holds its boundaries meaning that it remains clear and permits the deviation of systematic interrelationships without getting lost in the theory. |
|  | **LO1:** Does *‘parental sleep when their child is sick’* hold its boundaries through theoretical integration with other related concepts (e.g., in theories, models, or frameworks)?  **When to apply the code:**  **Yes:** *‘Parental sleep when their child is sick’* is discussed and theoretically integration in theories, models, or frameworks alongside other related concepts with evidence of conceptual boundaries.  **Partly:** *‘Parental sleep when their child is sick’* is discussed and to some extent theoretically integrated in theories, models, or frameworks alongside other related concepts with some evidence of conceptual boundaries.  **No:** *‘Parental sleep when their child is sick’* is not discussed or theoretically integrated in theories, models, or frameworks alongside other related concepts with no evidence of conceptual boundaries. |

**Supplementary 2: Literature overview**

| **Authors** | **Country** | **Aim** | **Method** | **Discipline** | **Context** | **Mothers** | **Fathers** | **Child condition** | **Child Age** |
| --- | --- | --- | --- | --- | --- | --- | --- | --- | --- |
| Adiga, Gupta, Khanna, Taly, and Thennarasu (2014) | India | To observe prevalence of sleep disturbance in cerebral palsy children in a specific age-group and its correlation with sleep disturbance in primary caregivers and other associated factors | Quantitative | Neurology | Tertiary research hospital | 50 | 0 | Cerebral palsy | 6-15 yrs |
| Al Maghaireh, Abdullah, Chong, Chua, and Al Kawafha (2017) | Jordan | To investigate the stressors and stress levels among Jordanian parents of infants in the NICU and their relationship to three factors: anxiety, depression, and sleep disturbance. | Quantitative | Nursing | Neonatal Intensive Care Unit | 155 | 155 | Neonatal | <28 - 37 wks |
| Albayrak, Biber, aliskan, and Levendoglu (2019) | Turkey | The aims of this study were to evaluate pain, care burden, depression level, sleep quality, fatigue, and quality of life. | Quantitative | Physical Medicine and Rehabilitation | University Medical Faculty | 101 | 0 | Cerebral palsy | ≤ 18 yrs |
| Angelhoff, Edéll-Gustafsson, and Mörelius (2015) | Sweden | To describe parents' perceptions of circumstances influencing their own sleep when living with a child enrolled in hospital-based home care services. | Qualitative | Nursing | Home | 11 | 4 | Endocrine system disease  Congenital heart defect  Cystic fibrosis  Esophageal atresia  Spinal muscular atrophy  Chromosome aberrations  Biliary tract atresia  Gastroschisis  Prematurely born with complications | 5mths-12 yrs |
| Angelhoff, Edéll-Gustafsson, and Mörelius (2017) | Sweden | To describe sleep quality and mood in parents accommodated with their sick child in a family-centred paediatric ward. | Quantitative | Nursing | Paediatric wards | 61 | 21 | Oncology  Diabetes  Neurology  Elective surgery  Emergency Infections | 0-18 yrs |
| Angelhoff, Askenteg, Wikner, and Edell-Gustafsson (2019) | Sweden | To explore and describe perceptions of sleep in parents of children <2 years old with AD, consequences of parental sleep loss, and what strategies the parents used to manage sleep loss to improve sleep. | Qualitative | Nursing | Paediatric Clinics | Not stated  Total parents = 12 | Not stated | Atopic Dermatitis | 0-2 yrs |
| Angelhoff, Sjølie, Mörelius, and Løyland (2020) | Norway  Sweden | To explore and describe how parents perceive their sleep when staying overnight with their sick child in hospital. A further aim was to explore and describe parents' perception of what circumstances influence their sleep in the hospital | Qualitative | Nursing | Paediatric wards | 18 | 4 | Leg fracture  Spinal surgery  Foot/heel surgery  Hip surgery  Urinary problems, treatment  Appendicitis  Myalgic encephalomyelitis  Migraine  Respiratory disorder  Abdominal pain  Limping  Leukaemia treatment | 4 wks – 16yrs |
| Bevan et al. (2019) | UK | To measure sleep quality and noise levels in hospital and compare these with the home environment. | Quantitative | Medicine | Paediatric wards | 16 | 0 | Medical  Acute conditions  Chronic conditions (e.g., cystic fibrosis, chronic renal disease) | 3-16 yrs |
| Boergers, Hart, Owens, Streisand, and Spirito (2007) | USA | Examined the associations between childhood sleep disorders and mothers' and fathers' sleep duration and daytime sleepiness. | Quantitative | Sleep | Paediatric sleep disorder clinic | Not stated  Total families = 107 | Not stated | Child sleep disorders | 2-12 yrs |
| Bourke-Taylor, Pallant, Law, and Howie (2013) | Australia | Investigated the frequency and impact of sleep interruption on mothers of school-aged children with developmental disabilities in Victoria, Australia. | Quantitative | Occupational Therapy  Nursing  Rehabilitation | Mail-out survey | 152 | 0 | Cerebral palsy  Developmental delay  Autism  Asperger syndrome  Intellectual disability  Language disorder  Epilepsy  Visual impairment  ADHD | School age, mean 9.5 yrs |
| Byars, Chini, Hente, Amin, and Boat (2020) | USA | The aims of this study with parents and their children with cystic fibrosis were to (1) assess prevalence of parent concerns for their own sleep and that of their children, (2) examine frequencies and developmental timing of specific sleep problems, (3) assess nocturnal sleep sufficiency for parents and children, (4) examine the relationships among overall sleep concern and specific sleep problems in parents and children, and (5) examine predictive models of sleep duration for caregivers and children. | Mixed Method | Behavioural Medicine and Psychology | Email, in-person or over the telephone | 71 | 8 | Cystic fibrosis | 1-18yrs |
| Cadart et al. (2018) | France | Evaluated the impact that having a child with sleep-disordered breathing had on their parents, including their own sleep quality. | Quantitative | Sleep | Sleep laboratory | 76 | 20 | Various rare and genetic conditions - most common were Down syndrome, Prader Willi syndrome, craniofaciostenosis, achondroplasia, neuromuscular diseases and polymalformation. | 1-18 yrs |
| Chamlin, Mattson, Frieden, and Williams (2005) | USA | To evaluate sleep disturbance and co-sleeping in young children with atopic dermatitis and their families | Quantitative | Dermatology | Paediatric dermatology practices | Not stated  Total families = 270 | Not stated | Atopic dermatitis | 0-6 yrs |
| Cheezum et al. (2013) | USA | To examine whether the caregivers of children with asthma living in Detroit experience sleep disruptions, the ways in which their sleep is interrupted, and the impact of any sleep disruption. | Mixed Method | Respiratory | Community based | 37 | 3 | Asthma | 6-12 yrs |
| Chu and Richdale (2009) | Australia | To determine whether children's sleep and/or or behaviour difficulties impacted on mothers' sleep and mothers' psychological wellbeing. | Quantitative | Psychology | Early intervention centres  Special Developmental Schools  Special schools  Support organisations | 46 | 0 | Developmental disabilities | 2-12 yrs |
| Cottrell and Khan (2005) | USA | To examine the relationship between parental sleep problems and other measures of parental adjustment to their children's chronic illness. | Quantitative | Neurology | Paediatric Neurology Clinic | Not stated  Total parents = 50 | Not stated | Epilepsy | Up to 5 years |
| Edell-Gustafsson, Angelhoff, Johnsson, Karlsson, and Mörelius (2014) | Sweden | To explore and describe how parents of preterm and/or sick infants in neonatal care perceive their sleep. | Qualitative | Nursing | Neonatal Intensive Care Unit | 8 | 4 | Neonatal | 7-46 days |
| Feeley et al. (2014) | USA | 1) Describe the sleep quality, caregiver burden, stress, depressive symptoms, and QOL in maternal caregivers of young children with BPD; 2) determine the relationships between sleep quality, caregiver burden, stress and QOL in maternal caregivers of children with BPD; 3) examine the influences of QOL on age, depressive symptoms, education, marital status, sleep quality, caregiver burden, and stress 4) determine if stress mediates the relationship between sleep quality, caregiver burden and QOL in maternal caregivers of young children with BPD. | Quantitative | Nursing | Paediatric clinic | 61 | 0 | Bronchopulmonary Dysplasia (BPD) | 4-36 mths |
| Feeley et al. (2019) | USA | The purpose of this study was to explore caregivers' description of their experience of night-time sleep. | Mixed Method | Nursing/ Diabetes/ Medicine | Type 1 Diabetes Camp | 21 | 1 | Type 1 Diabetes | 10-18yrs |
| Feeley et al. (2021) | USA | The overall purpose of this study was to explore associations between parent and child sleep in school-age children (aged 6-12 years) with Type 1 Diabetes using questionnaires and actigraphy an describe sleep in the dyad. The study also examined the relationship between sleep and the symptoms of stress and depressive symptoms in parents and anxiety, fatigue, and A1C in children. | Quantitative | Nursing | Paediatric clinic | 15 | 3 | Type 1 Diabetes | 6-12yrs |
| Filiz, Keles, Akbulut, Isik, and Kara (2020) | Spain | To examine sleep patterns and sleep disturbances of children with food allergy and their mothers. | Quantitative | Paediatrics allergy/ gastroenterology/ psychiatry | Paediatric Allergy and Gastroenterology Clinics | 71 | 0 | Food Allergy | 2-10 yrs |
| Franck et al. (2014) | UK | Compare the sleep quality and quantity of parents who were accommodated at Ronald McDonald House with that of parents who slept at their child's bedside. | Mixed Method | Nursing | Paediatric Intensive Care Unit/ Ronald McDonald House | 27 | 8 | Not stated | 0-16 yrs |
| Gallagher, Phillips, and Carroll (2009) | UK | The aims were to examine: a) sleep patterns in parents of children with and without developmental disabilities (DD), b) the associations between stress, child problem behaviour, social support, and sleep quality in parents of children with DD, c) whether unhealthy behaviours, such as smoking, alcohol, and caffeine consumption, and BMI account for any associations | Quantitative | Psychology | Respective syndrome associations and family support groups | 53 | 14 | Developmental disabilities | 3-19 yrs |
| Gedaly-Duff, Lee, Nail, and Johnson (2006) | USA | To determine the feasibility of collecting symptom data at home from school-age children with acute lymphoblastic leukemia and from their fathers and mothers and to obtain initial descriptions of pan, sleep disturbance, and fatigue experienced by the family members at home | Quantitative | Nursing | Home | 7 | 6 | Acute lymphoblastic leukemia | 8-16 yrs |
| Goldman, Bichell, Surdyka, and Malow (2012) | USA | 1) obtain additional objective and subjective data on sleep in children/adolescents with Angelman syndrome 2) relate these sleep patterns to the primary parental caregiver’s sleep, daytime sleepiness, and stress. | Quantitative | Neurology | Research centre and Home | 16 | 0 | Angelman syndrome | 6-12 yrs |
| Hansen, Weissbrod, Schwartz, and Patrick Taylor (2012) | USA | The primary aim of this exploratory study was to examine the relationship between fathers' diabetes care and paternal psychological functioning. | Quantitative | Paediatrics | Home | 82 | 43 | Type 1 Diabetes | 7-14 yrs |
| Heaton, Noyes, Sloper, and Shah (2006) | UK | Examine the temporal organisation of the care routines for technology-dependent children living at home and the effects of the regimes on the families. | Quantitative | Nursing/ Social Policy | Home | 34 | 12 | Neuro-disability, respiratory, renal, neuro-degenerative gastrointestinal, cardiac, metabolic, congenital abnormality, haematological | 16mth – 19 yrs |
| Herbert, Monaghan, Cogen, and Streisand (2014) | USA | This study investigated sleep characteristics among parents of children with Type 1 Diabetes and relationships among parent's sleep quality, hypoglycaemia worry and diabetes self-efficacy. | Mixed Method | Endocrinology | Home | 120 | 14 | Type 1 Diabetes | 1-6 yrs |
| Jaser et al. (2017) | USA | Explored relationships between parental well-being, fear of hypoglycaemia, nocturnal caregiving, and children's sleep patterns. | Quantitative | Endocrinology | Registry data | Not stated  Total parents = 515 | Not stated | Type 1 Diabetes | 2-12 yrs |
| Johnson et al. (2018) | USA | We explored the sleep of caregivers of teenagers with asthma and investigated the association of socioeconomic status and various psychosocial stressors will be associated with shorter sleep duration and poorer sleep quality. | Quantitative | Respiratory | Emergency department | 87 | 11 | Asthma | 13-19 yrs |
| Keilty, Cohen, Spalding, Pullenayegum, and Stremler (2018) | Canada | The aim of this study was to compare sleep in family caregivers of children who depend on medical technology with that of family caregivers of healthy, same-aged children. | Quantitative | Medical Technology/ Rehabilitation | Home | 35 | 7 | Cardiac, Genetic/metabolic, neurological, respiratory, musculoskeletal, neurocognitive delay/impairment | 12 mths – 18 yrs |
| Larson et al. (2012) | USA | To explore the effect of paediatric epilepsy on child sleep, parental sleep and fatigue, and parent-child sleeping arrangements, including room sharing and co-sleeping. | Quantitative | Neurology | Home | 99 | 6 | Absence epilepsy, Doose syndrome, Lennox-Gastaut, West syndrome, benign rolandic epilepsy and electrical status epilepticus in sleep and other forms | 2-10 yrs |
| Lee, Lee, Rankin, Weiss, and Alkon (2007) | USA | To describe Chinese-American parents' sleep disturbances and fatigue in relation to their stress levels, resulting from the hospitalisation of their infants in intensive care units. | Quantitative | Nursing | Intensive Care Unit | 30 | 25 | Premature, respiratory distress, sepsis, and congenital heart disease | 3 days – 1 yr |
| Lee and Kimble (2009) | USA | Explore relationships between impaired sleep and well-being in mothers with low-birth weight infants in the neonatal intensive care unit. | Quantitative | Nursing | Neonatal Intensive Care Unit | 20 | 0 | Low birth weight | 24-36 wks |
| Lee and Hsu (2012) | USA | The purpose was to examine the relations between sleep, stress, depression, fatigue and HRQOL among mothers with a LBW infant in the NICU during early postpartum. | Quantitative | Nursing | Home | 55 | 0 | Low birth weight | 5-10 days |
| Liu, Yin, Zhu, Zhang, and Sheng (2021) | China | The study aims to investigate the correlation between family resilience, sleep quality, and depression in parents of children with epilepsy. | Quantitative | Neurology | Hospital | 90 | 67 | Epilepsy | 0-18yrs |
| Lopez-Wagner, Hoffman, Sweeney, Hodge, and Gilliam (2008) | USA | Compared parent's reports of their own sleep problems for both parents of typically developing children and parents of children with autism. Examined the relation between children's sleep problems and those of their parents for these groups. | Quantitative | Psychology | Research Centre/ Home | Not stated  Total parents: 106 | Not stated | Autism | 4-16 yrs |
| Mcbean and Schlosnagle (2016) | USA | Aimed to replicate the findings that parents of children with special healthcare needs report poorer sleep quality than parents of typically developing children, and further examined how sleep is related to general health and memory. | Quantitative | Medicine | Online | 97 | 0 | Special health care needs | 1 mth – 31 yrs |
| McCann (2008) | Australia | The purpose was to a) describe the sleep experiences of parents staying with their children and b) identify variables that may influence the length and quality of parents sleep | Mixed Method | Nursing | Paediatric Hospital wards | 83 | 19 | Medical, surgical, oncology | 10 days – 15 yrs |
| McLoone, Wakefield, Yoong, and Cohn (2013) | Australia | The purpose of this study was to provide the first prevalence estimates of self-reported sleep quantity and quality among parents accommodated on the paediatric ward, compared to parents of age-matched controls. | Mixed Method | Oncology | Paediatric Hospital – inpatients | 39 | 13 | Leukemia, Lymphoma, Solid tumours | Mean = 7.4 (5.3) |
| Meltzer and Mindell (2006) | USA | Examine the sleep patterns and causes of sleep disturbances in caregivers of children with and without chronic illnesses and to determine whether sleep mediates the relationship between a child's chronic illness and daytime functioning in caregivers. | Quantitative | Medicine  Paediatric  Psychology | Telephone | 71 | 0 | Ventilator dependency, Cystic fibrosis, and healthy controls | 3-14 yrs |
| Meltzer (2008) | USA | Examine sleep quality and sleep-wake patterns in parents of children with and without autism spectrum disorders. | Quantitative | Paediatrics | Home | 20 | 12 | Autism spectrum disorder,  Asperger’s,  Pervasive developmental disorder | 4-10 yrs |
| Meltzer, Boroughs, and Downes (2010) | USA | The purpose of this exploratory study was to examine whether there is a relationship between home-care nursing coverage and caregiver functioning, in particular sleep, mood, and daytime functioning. | Quantitative | Medicine  Paediatric  Psychology | Telephone | 29 | 7 | Neuromuscular  Nervous system  Chronic lung disease Congenital anomaly | 3-14 yrs |
| Meltzer, Davis, and Mindell (2012) | USA | The first aim was to examine whether the previous night of sleep in hospital differed from typical sleep at home. | Quantitative | Psychology  Nursing  Sleep | Paediatric Hospital | 52 | 6 | Non intensive care paediatric inpatients | 8-21 years |
| Meltzer, Sanchez-Ortuno, Edinger, and Avis (2015) | USA | To explore differences in sleep between parents of ventilator-assisted children and parents of health children, as well as whether there is a relationship between objective sleep patterns (including night-to-night sleep instability) and health-related quality of life in parents of children with and without ventilator-assistance. | Quantitative | Psychology  Sleep | Home | 42 | 14 | Ventilator-assisted children | 4-17 yrs |
| Meltzer and Booster (2016) | USA | To examine sleep patterns and sleep disturbances in caregivers of children with chronic illness. | Quantitative | Psychology | Home | 145 | 35 | Atopic dermatitis  Asthma  Ventilator assistance | Average age 8.7 years (SD=5.1) |
| Meltzer and Pugliese (2017) | USA | The purpose of this study was to characterise sleep in young children (one to four years) with and without asthma and their parents. | Quantitative | Paediatrics/ Neuropsychology | Online | 105  27: Described as other | 68 | Asthma | 1-4yrs |
| Mihaila and Hartley (2016) | USA | Explored the impact of parental sleep quality on the experience of behaviour problems by children with autism spectrum disorder. | Quantitative | Developmental Disability | Online | 176 | 176 | Autism | 5-12 yrs |
| Moore, David, Murray, Child, and Arkwright (2006) | UK | To compare the impact of caring for a child with atopic eczema vs. asthma on parent's sleep and well-being. | Quantitative | Eczema  Asthma | Paediatric Hospital | 55 | 37 | Eczema  Asthma | Up to 16 yrs |
| Mörelius and Hemmingsson (2013) | Sweden | To study whether sleep problems and need for night-time attention among children with physical disabilities are associated with perceived parental health, headache, psychological exhaustion, pain due to heavy lifting, night-time wakefulness, and disrupted sleep. | Quantitative | Physical disabilities | Rehabilitation centre | 377 | 377 | Cerebral palsy Spinal bifida Muscular dystrophy | 1-16 yrs |
| Nassery and Landgren (2018) | Sweden | The aim of this study was to explore parents' experiences of sleep and rest while admitted to hospital together with their ill child. | Qualitative | Nursing | Paediatric wards | 12 | 5 | Infection  Cariology  Intensive care Neonatal care Endocrinology General care | 1 mth – 15 yrs |
| Neu, Matthews, and King (2014) | USA | The aim of this study was to explore maternal perception of their sleep quality during maintenance treatment for their child's Acute Lymphoblastic Leukemia, and to discover what sleep strategies mothers used to attain sleep, and/or cope with lack of sleep | Qualitative | Nursing | Oncology  Nursing  Paediatrics | 20 | 0 | Acute lymphoblastic leukemia | 3.5-12yrs |
| Nozoe et al. (2016) | Brazil | The aim of this study was to evaluate the sleep quality of caregiver-mothers of sons with Duchenne muscular dystrophy and factors that are associated with their sleep quality. | Quantitative | Psychobiology | Neuromuscular Centre | 32 | 0 | Duchene muscular dystrophy patients | 8-15 yrs |
| Ortiz-Rubio et al. (2021) | Spain | The study was conducted to explore the degree to which caregiver burden is associated with sleep quality in parents of children with autism spectrum disorder, and to determine a statistically valid cutoff score for the Caregiver Burden Inventory (CBI) in order to identify parents of risk of poor sleep. | Quantitative | Physiotherapy | Special childcare centre | 100 | 16 | Autism Spectrum Disorder | 11.22 yrs ± 6.05 |
| Pouraboli, Poodineh, and Jahani (2019) | Iran | This study aimed to determine the effect of relaxation techniques on anxiety, fatigue, and sleep quality of parents of children with leukemia under chemotherapy in South East Iran in 2015. | Quantitative | Nursing | Teaching Hospital | Not stated  Total parents: 120 | Not stated | Leukemia | Not stated |
| Ramirez et al. (2019) | USA  Data from UK | To compare sleep disturbances over time between mothers of children with and without atopic dermatitis and to determine whether these disturbances are associated with the child's disease severity and the child’s sleep disturbances | Quantitative | Dermatology | Population Based birth cohort | 4767 | 0 | Atopic Dermatitis | 0-11yrs |
| Ridolo et al. (2015) | Italy | Evaluated the presence of disrupted sleep in parents of children with atopic disorders, and its relationship with clinical features and the presence of disturbed sleep. | Quantitative | Allery  Respiratory | Paediatric Allery Units | 66 | 24 | Asthma  Rhinitis  Atopic dermatitis | 7.7 yrs ± 4.3 |
| Safa, Khalilzadeh, Talischi, and Alizadeh (2012) | Iran | Evaluating the correlation between depression-anxiety and sleep quality in mothers of children suffering from cystic fibrosis and asthma hospitalised. | Quantitative | Respiratory | Hospital | 48 | 0 | Asthma  Cystic Fibrosis | Not stated |
| Shaki, Goldbart, Daniel, Fraser, and Shorer (2011) | Israel | To evaluate the effects of paediatric epilepsy on sleep in parents of epileptic children. | Quantitative | Epilepsy | Paediatric Departments  Emergency room | 26 | 13 | Epilepsy | ≤ 18 yrs |
| Stremler, Dhukai, Wong, and Parshuram (2011) | Canada | To describe factors affecting the sleep of parents of critically ill children and to determine strategies used to improve their sleep. | Qualitative | Nursing | Hospital | 74 | 44 | Acute illness/ trauma Exacerbation of chronic illness Planned surgery | Under 18 yrs |
| Stremler et al. (2014) | Canada | To describe sleep quantity, sleep patterns, fatigue, and sleepiness for parents of critically ill hospitalised children. | Quantitative | Nursing | Paediatric Intensive Care Unit | 74 | 44 | Planned surgery Acute illness/trauma Chronic illness | <1 yr - >13 yr |
| Stickland, Clayton, Sankey, and Hill (2016) | UK | To study the experiences of children and parents during hospital admissions. | Qualitative | Nursing | Hospital | 16 | 1 | Acute illnesses predominantly respiratory tract infections.  Chronic illnesses including leukaemia and cystic fibrosis | 3-12 yrs |
| Tietze, Zernikow, Michel, and Blankenburg (2014) | Germany | Systematically assessed how much a child's sleep disturbance affects parental sleep and quality of life. | Quantitative | Neurology | Hospital | 212 | 193 | Severe psychomotor impairment | 1mth – 25 yrs |
| Ullas, Maharana, Metri, Gupta, and Nagendra (2021) | India | This study assessed the impact of a 1-month yoga intervention on anxiety, depression, stress and sleep quality in mothers of children with intellectual disabilities. | Quantitative | Alternative therapy | Not clearly stated | 53 | 0 | Intellectual disability | Not stated |
| Varma, Conduit, Junge, and Jackson (2020) | Australia | The current study examined sleep and mood associations in parents of children with sleep disturbances across a sample of typically developing children and children with neurodevelopmental disorders. | Quantitative | Health and Biomedical Sciences/ Sleep/ Brain and Mental Health/ Psychology | Local community | 85 | 0 | Neurodevelop-mental disorders | 2-12 yrs |
| Wayte, McCaughey, Holley, Annaz, and Hill (2012) | UK | To study the relationship between sleep problems in children with CP and maternal sleep quality and depression. | Quantitative | Medicine  Psychology | Disability Database | 40 | 0 | Quadriplegic Hemiplegic  Diplegic  Ataxic (8%) | Mean age 7.8 (SD 2.4) |
| Wright, Tancredi, Yundt, and Larin (2006) | Canada | The purpose of this study was to describe sleep patterns, concerns, associated factors, and strategies to address sleep issues in children with and without physical disabilities | Mixed Method | Physical therapy | Children’s Treatment Centre | 177 | 64 | Cerebral palsy  Spina Bifida Muscular Dystrophy Developmental delay | 0-1 6 (4), 2-5 55 (31), 6-12 69 (39), 13+ 43 (24%) |
| Wright (2010) | Canada | To describe and gain an understanding of the sleep characteristics of children receiving treatment for cancer and their caregivers using a mixed methods concurrent triangulation design. | Mixed Method | Physical therapy | Children’s Hospital | Not stated  Total parents = 35 | Not stated | Acute lymphoblastic leukemia  Sarcoma  CNS tumour  Other oncological diagnoses 2 (6%) | Cancer 7.9 (4.8), |
| Yang et al. (2020) | China | The purpose of the study was to evaluate sleep quality in the parents of children with epilepsy as well as their symptoms of anxiety and depression in Southern China. | Quantitative | Neurology | Paediatrics/ Neurology Hospital | 158 | 76 | Epilepsy | 0-18yrs |
| Yılmaz et al. (2008) | Turkey | The aim was to compare sleep quality and depression-anxiety parameters in mothers of children with cystic fibrosis, asthma, and healthy controls. | Quantitative | Allergy Pulmonology  Nephrology | Paediatrics Outpatient Department | 83 | 0 | Asthma  Cystic Fibrosis (CF) | Asthma mean 8.1 ± 2.9yrs  CF mean 6.1 ± 4.6years |
| Yilmaz and Alemdar (2020) | Turkey | The aim of the study was to determine the correlation between care burden and physical activity, quality of life, and sleep quality of mothers with disabled children. | Quantitative | Nursing | Private Rehabilitation Centres | 165 | 0 | Disability | 6-12yrs |
| Yuksel et al. (2007) | Turkey | The aim of this study was to evaluate sleep quality in asthmatic children and their mothers as well as the status of anxiety-depression in the mothers | Quantitative | Respiratory | Paediatrics | 75 | 46 | Asthma | 7-16 yrs |
| Yuwen et al. (2016) | USA | Describe daily sleep patterns, sleep quality and sleep hygiene in 2-5-year-old children newly diagnosed with Juvenile Idiopathic Arthritis and their parents in comparison with typically developing children and parents. | Quantitative | Pulmonary Sleep medicine | Paediatric Rheumatology clinic | 13 | 0 | Juvenile Idiopathic Arthritis | 2-5 yrs |
| Zupanec, Jones, and Stremler (2010) | Canada | This study also described sleep habits, sleep disturbance, and fatigue of parents of children and adolescents with ALL and determined if relationships existed between parent and child sleep disturbance and fatigue. | Mixed Method | Nursing | Acute lymphoblastic leukaemia | 49 | 9 | Acute lymphoblastic leukaemia | 4-18 yrs |

**Supplementary 3: Table of references**

| **Point** | **Point in text** | **Additional references** |
| --- | --- | --- |
| **1** | Parents of typically developed children  Controls  Parents of healthy children | (Chu & Richdale, 2009; Gallagher et al., 2009; Lopez-Wagner et al., 2008; Mcbean & Schlosnagle, 2016; Meltzer & Booster, 2016; Meltzer & Moore, 2008; Yuwen et al., 2016)  (Albayrak et al., 2019; Filiz et al., 2020)  (Albayrak et al., 2019; Wright, 2010) |
| **2** | Part of the research aims for 26 out of 74 articles. | (Albayrak et al., 2019; Angelhoff et al., 2017; Bevan et al., 2019; Cadart et al., 2018; Feeley et al., 2014; Franck et al., 2014; Gallagher et al., 2009; Herbert et al., 2014; Johnson et al., 2018; Liu et al., 2021; Mcbean & Schlosnagle, 2016; McCann, 2008; McLoone et al., 2013; Meltzer & Moore, 2008; Mihaila & Hartley, 2016; Neu et al., 2014; Nozoe et al., 2016; Ortiz-Rubio et al., 2021; Pouraboli et al., 2019; Safa et al., 2012; Ullas et al., 2021; Wayte et al., 2012; Yang et al., 2020; Yilmaz & Alemdar, 2020; Yılmaz et al., 2008; Yuksel et al., 2007; Yuwen et al., 2016). |
| **3** | Disturbance  Disruption  Interruption  Fragmented  Nocturnal awakenings  Arousal  Wake after sleep onset | (Filiz et al., 2020; Lee & Hsu, 2012; Ortiz-Rubio et al., 2021; Ramirez et al., 2019; Zupanec et al., 2010)  (C. Angelhoff et al., 2019; Angelhoff et al., 2015; Bevan et al., 2019; Cheezum et al., 2013; Edell-Gustafsson et al., 2014; Franck et al., 2014; Goldman et al., 2012; Heaton et al., 2006; Keilty et al., 2018; Lee & Hsu, 2012; Lee & Kimble, 2009; McCann, 2008; McLoone et al., 2013; Meltzer & Booster, 2016; Meltzer et al., 2010; Nassery & Landgren, 2018; Neu et al., 2014; Stremler et al., 2011; Tietze et al., 2014)  (Angelhoff et al., 2017; Chu & Richdale, 2009; McLoone et al., 2013)  (Feeley et al., 2019; Gedaly-Duff et al., 2006; Stremler et al., 2014)  (C. Angelhoff et al., 2019; Angelhoff et al., 2015; Angelhoff et al., 2017; Angelhoff, Edéll-Gustafsson, & Mörelius, 2019)  (Keilty et al., 2018; Stremler et al., 2014)  (Gedaly-Duff et al., 2006; Goldman et al., 2012; Lee & Kimble, 2009; Meltzer et al., 2015; Yuwen et al., 2016) |
| **4** | Noise  Light  Sleeping accommodation  Interruptions from healthcare professionals | (Bevan et al., 2019; Edell-Gustafsson et al., 2014; Feeley et al., 2014; Franck et al., 2014; McCann, 2008; McLoone et al., 2013; Meltzer et al., 2012; Nassery & Landgren, 2018; Stickland et al., 2016; Stremler et al., 2011)  (McLoone et al., 2013; Stickland et al., 2016; Stremler et al., 2011)  (Al Maghaireh et al., 2017; McLoone et al., 2013)  (McLoone et al., 2013; Stremler et al., 2011) |
| **5** | Parents caring for a sick child were found to sleep less than 7 hours per day. | (Feeley et al., 2019; Feeley et al., 2021; Herbert et al., 2014; Jaser et al., 2017; Johnson et al., 2018; Keilty et al., 2018; Lee & Hsu, 2012; Lee & Kimble, 2009; McCann, 2008; McLoone et al., 2013; Meltzer & Booster, 2016; Meltzer et al., 2015; Stremler et al., 2014) |
| **6** | Sleep duration  Lack of sleep  Sleep loss  Sleep less  Sleep quantity  Sleep efficiency  Total sleep time | (Adiga et al., 2014; Angelhoff et al., 2015; Angelhoff et al., 2017; Boergers et al., 2007; Cheezum et al., 2013; Feeley et al., 2019; Franck et al., 2014; Herbert et al., 2014; Jaser et al., 2017; Johnson et al., 2018; Keilty et al., 2018; Larson et al., 2012; McLoone et al., 2013; Meltzer & Booster, 2016; Meltzer & Mindell, 2006; Meltzer et al., 2015; Ridolo et al., 2015; Safa et al., 2012; Shaki et al., 2011; Stickland et al., 2016; Stremler et al., 2014; Tietze et al., 2014; Wright, 2010; Yılmaz et al., 2008; Yuksel et al., 2007; Yuwen et al., 2016)  (Chu & Richdale, 2009; Edell-Gustafsson et al., 2014; Heaton et al., 2006; Jaser et al., 2017; McCann, 2008; Neu et al., 2014; Stickland et al., 2016)  (C. Angelhoff et al., 2019; Bevan et al., 2019; Chamlin et al., 2005; Moore et al., 2006)  (Meltzer, 2008; Wright, 2010; Wright et al., 2006)  (Cheezum et al., 2013; Franck et al., 2014; Larson et al., 2012)  (Boergers et al., 2007; Lopez-Wagner et al., 2008; Nozoe et al., 2016)  (Bevan et al., 2019; Goldman et al., 2012; Lee & Hsu, 2012; Yuwen et al., 2016) |
| **7** | The most frequently used tool in this dataset was the Pittsburgh Sleep Quality Index (PSQI) used in 34 of the 74 research studies | (Adiga et al., 2014; Albayrak et al., 2019; Cadart et al., 2018; Chu & Richdale, 2009; Cottrell & Khan, 2005; Feeley et al., 2021; Feeley et al., 2014; Filiz et al., 2020; Gallagher et al., 2009; Hansen et al., 2012; Herbert et al., 2014; Jaser et al., 2017; Keilty et al., 2018; Larson et al., 2012; Liu et al., 2021; Lopez-Wagner et al., 2008; Mcbean & Schlosnagle, 2016; Meltzer, 2008; Meltzer & Booster, 2016; Meltzer & Mindell, 2006; Nozoe et al., 2016; Ortiz-Rubio et al., 2021; Pouraboli et al., 2019; Ridolo et al., 2015; Safa et al., 2012; Shaki et al., 2011; Tietze et al., 2014; Ullas et al., 2021; Varma et al., 2020; Wayte et al., 2012; Yang et al., 2020; Yilmaz & Alemdar, 2020; Yılmaz et al., 2008; Yuksel et al., 2007) |
| **8** | Depression  Anxiety  Stress  Sleepiness  Parental adjustment/impact to their child’s condition  Burden  Quality of life  Fatigue | (Albayrak et al., 2019; Cadart et al., 2018; Chu & Richdale, 2009; Feeley et al., 2014; Filiz et al., 2020; Liu et al., 2021; Meltzer & Booster, 2016; Meltzer & Mindell, 2006; Safa et al., 2012; Tietze et al., 2014; Ullas et al., 2021; Yılmaz et al., 2008; Yuksel et al., 2007)  (Chu & Richdale, 2009; Ullas et al., 2021)  (Chu & Richdale, 2009; Gallagher et al., 2009; Ullas et al., 2021)  (Cadart et al., 2018; Meltzer & Booster, 2016; Tietze et al., 2014)  (Cottrell & Khan, 2005; Herbert et al., 2014  (Albayrak et al., 2019; Feeley et al., 2014; Ortiz-Rubio et al., 2021; Yilmaz & Alemdar, 2020)  (Albayrak et al., 2019; Feeley et al., 2014; Tietze et al., 2014)  (Albayrak et al., 2019; Larson et al., 2012; Meltzer & Booster, 2016; Meltzer & Mindell, 2006) |
| **9** | Wrist actigraphy was the most used objective measure  Actigraphy was also often recommended for future studies to remove biased reporting | (Bevan et al., 2019; Feeley et al., 2021; Franck et al., 2014; Gedaly-Duff et al., 2006; Goldman et al., 2012; Keilty et al., 2018; Lee & Hsu, 2012; Lee & Kimble, 2009; Lee et al., 2007; Meltzer, 2008; Meltzer et al., 2015; Stremler et al., 2014; Yuwen et al., 2016)  (Angelhoff et al., 2017; Feeley et al., 2019; Herbert et al., 2014; Keilty et al., 2018; Lee & Hsu, 2012; Lee & Kimble, 2009; Mcbean & Schlosnagle, 2016; McLoone et al., 2013; Meltzer & Booster, 2016; Meltzer et al., 2010; Meltzer & Mindell, 2006; Meltzer & Pugliese, 2017; Mihaila & Hartley, 2016; Tietze et al., 2014; Wayte et al., 2012; Yang et al., 2020; Zupanec et al., 2010) |
| **10** | 34 of the 56 studies that included quantitative methods included fewer than 100 participants  Eight studies reported power calculations | (Adiga et al., 2014; Angelhoff et al., 2017; Bevan et al., 2019; Cadart et al., 2018; Chu & Richdale, 2009; Cottrell & Khan, 2005; Feeley et al., 2021; Feeley et al., 2014; Filiz et al., 2020; Gallagher et al., 2009; Gedaly-Duff et al., 2006; Goldman et al., 2012; Heaton et al., 2006; Johnson et al., 2018; Keilty et al., 2018; Lee & Hsu, 2012; Lee & Kimble, 2009; Lee et al., 2007; Mcbean & Schlosnagle, 2016; Meltzer, 2008; Meltzer et al., 2010; Meltzer et al., 2012; Meltzer & Mindell, 2006; Meltzer et al., 2015; Moore et al., 2006; Nozoe et al., 2016; Ridolo et al., 2015; Safa et al., 2012; Shaki et al., 2011; Ullas et al., 2021; Varma et al., 2020; Wayte et al., 2012; Wright, 2010; Yılmaz et al., 2008; Yuwen et al., 2016)  (Meltzer, 2008; Meltzer et al., 2010; Nozoe et al., 2016; Ortiz-Rubio et al., 2021; Ridolo et al., 2015; Shaki et al., 2011; Yilmaz & Alemdar, 2020; Yuwen et al., 2016) |
| **11** | Longitudinal research  Support/information provision  Assessing parents’ sleep  Fathers  Larger samples | (Albayrak et al., 2019; Johnson et al., 2018; Keilty et al., 2018; Lee et al., 2007; Liu et al., 2021; McLoone et al., 2013; Meltzer & Booster, 2016; Meltzer et al., 2010; Meltzer & Mindell, 2006; Meltzer et al., 2015; Ortiz-Rubio et al., 2021; Safa et al., 2012; Stremler et al., 2014; Stremler et al., 2011)  (Cheezum et al., 2013; Feeley et al., 2021; Feeley et al., 2014; Larson et al., 2012; McCann, 2008; Ortiz-Rubio et al., 2021; Pouraboli et al., 2019; Yilmaz & Alemdar, 2020)  (Angelhoff et al., 2015; Feeley et al., 2021)  (Meltzer, 2008; Meltzer & Booster, 2016; Meltzer et al., 2010; Mörelius & Hemmingsson, 2013; Yilmaz & Alemdar, 2020; Yılmaz et al., 2008)  (Filiz et al., 2020; Johnson et al., 2018; Lee & Hsu, 2012; Lee & Kimble, 2009; Lee et al., 2007; Meltzer, 2008; Ullas et al., 2021; Wayte et al., 2012) |
