# Supplementary material for: Parental sleep when their child is sick: A phased principle‐based concept analysis
Source: J Sleep Res. 2022 Apr 25;31(5):e13575. doi: 10.1111/jsr.13575 (PMC9786861; doi:10.1111/jsr.13575)
Supplement: Supplementary file 2 — Figure 1 [file JSR-31-e13575-s002.docx]

Additional records identified through other sources
(n = 45)

Records identified through database searching
(n = 501)

## Identification

Records after duplicates removed
(n = 383)

## Screening

Records excluded
(n = 294)

Records screened
(n = 383)

## Eligibility

Full-text articles assessed for eligibility
(n = 89)

Full-text articles excluded, with reasons
(n = 15)

## Included

Studies included
(n = 74)

**Figure 1** PRISMA flow diagram of the screened studies
